# Strengthening systems to monitor antibiotics. An action oriented approach

**DOI:** 10.12688/wellcomeopenres.24568.2

**Published:** 2026-09-24

**Authors:** Olafur Valsson, Elizabeth Tayler, Freddy Eric Kitutu, Jennifer Bonnah, Javiera Cornejo, Alejandro Dorado Garcia, Anahí Dreser, Aitziber Echeverria, Sabiha Y. Essack, Stanley Fenwick, Walter Fuller, Nada Hanna, Jennifer Hegewisch-Taylor, Bronwen Holloway, Morgan Jeannin, Jyoti Joshi, Henson Kainga, Amit Khurana, Shaffi Fazaludeen Koya, D. G. Joakim Larsson, Angkana Lekagul, Jane Lwoyero, Sophie Masika, Ana L. P. Mateus, Collins Mitambo, Nyambura Moremi, Dishon Muloi, Mwapu Ndahi, Mary Nkansa, Joseph Nkhoma, Sharon Odeo, Pascale Ondoa, Mike Sharland, Renata Tigulini de Souza Bogo, Susanna Sternberg-Lewerin, Marie Verhaegen, David Verner-Jeffreys, J Scott Weese, Kaunda Yamba, S. M. Sabrina Yesmin, Janet Midega, Clare I R Chandler

**Affiliations:** 1 Holaholar ehf, Akureyri, Horgarsveit, 604, Iceland; 2 Antibiotic Policy Group, City St Georges University, London, UK; 3 Department of Pharmacy, Makerere University School of Health Sciences, Kampala, Central Region, Uganda; 4 Food and Drugs Authority, Accra, Ghana; 5 Center for antimicrobial stewardship in aquaculture, University of Chile Faculty of Veterinary Science, Santiago, Chile; 6 Food and Agriculture Organization of the United Nations, Rome, Italy; 7 Center for Health Systems Research, National Institute of Public Health, Cuernavaca, Morelos, Mexico; 8 United Nations Environment Programme, Geneva, Switzerland; 9 Antimicrobial Research Unit, School of Health Sciences, University of KwaZulu-Natal, Durban, South Africa; 10 Mott Macdonald, London, UK; 11 Regional Office for Africa, World Health Organization, Brazzaville, Congo; 12 Faculty of Public Health and Policy, London School of Hygiene and Tropical Medicine, London, UK; 13 ReAct - Department of Medical Sciences, Uppsala University, Uppsala, Sweden; 14 World Organisation for Animal Health (WOAH), Paris, France; 15 International Centre for Antimicrobial Resistance Solutions, Copenhagen, Denmark; 16 Department of Veterinary Epidemiology and Public Health, Faculty of Veterinary Medicine, Lilongwe University of Agriculture and Natural Resources, Lilongwe, Central Region, Malawi; 17 Sustainable Food Systems programme, Centre for Science and Environment, New Delhi, Delhi, India; 18 World Health Organization. Regional Office for the Eastern Mediterranean., Cairo, Egypt; 19 Department of Infectious Diseases, Institute of Biomedicine, The Sahlgrenska Academy at the University of Gothenburg, Gothenburg, Sweden; 20 Centre for Antibiotic Resistance Research in Gothenburg (CARe), Gothenburg, Sweden; 21 International Health Policy Program Foundation, Nonthaburi, Thailand; 22 World Organisation for Animal Health, Sub-Regional Representation for Eastern Africa, Nairobi, Kenya; 23 World Federation for Animals, Boston, USA; 24 Ministry of Health, Lilongwe, Malawi; 25 National Public Health Laboratory, Dar es Salam, Tanzania; 26 International Livestock Research Institute, Nairobi, Nairobi County, Kenya; 27 Federal Ministry of Livestock Development, Abuja, Nigeria; 28 Fisheries Commission, Accra, Ghana; 29 Central Veterinary Laboratory, Lilongwe, Malawi; 30 Ecumenical Pharmaceutical Network, Nairobi, Kenya; 31 The Global Fund to Fight AIDS Tuberculosis and Malaria, Grand-Saconnex, Geneva, Switzerland; 32 Antibiotic Policy Group, Institute of Infection and Immunity, City St George's, University of London, London, UK; 33 Brazilian Ministry of Health, Asa Norte, Brazil; 34 Dept of Animal Biosciences, Swedish University of Agricultural Sciences, Uppsala, Sweden; 35 WorldFish, Bayan Lepas, Penang, Malaysia; 36 University of Guelph, Ontario Veterinary College, Department of Pathobiology, Guelph, Ontario, Canada; 37 ReAct Africa, Lusaka, Zambia; 38 Directorate General of Drug Administration, Dhaka, Bangladesh; 39 Wellcome Trust, London, England, UK; 40 Department of Global Health & Development, London School of Hygiene & Tropical Medicine, London, England, UK

**Keywords:** Antibiotic use, monitoring, surveillance, antimicrobial resistance, one health

## Abstract

**Background:**

Antibiotics have become lynchpins of our modern systems of healthcare, animal health and agriculture. Monitoring the types and volumes of antibiotics distributed, used and discharged across these systems is critical to provide evidence for action. With limited resources, implementation of multiple tools and recommendations for antibiotic monitoring is challenging. This paper sets out principles and case study illustrations for establishing systems to monitor antibiotic supply and use in resource-constrained settings.

**Methods:**

A technical working group and wider reference group drew together expertise and experience from 71 individuals representing different countries, continents and One Health domains, to establish the value of monitoring antibiotics, review tools and guidelines, share experiences and generate principles for prioritising action. This included a literature review, in person workshop, online meetings and collaborative writing between August 2024 and May 2025.

**Results:**

The literature search identified two types of outputs: a) antibiotic monitoring and surveillance tools (covering data collection-oriented and residue control tools), and b) guidelines, standards, and mapping/assessment tools for monitoring antibiotics. The group identified multiple purposes for establishing a coordinated system for monitoring antibiotic use across human, animal, plant and environmental domains at country level, including ensuring access, and informing efforts to mitigate antimicrobial resistance (AMR). Tools to monitor use closer to end-user level were less standardised and often more complex. Proposed steps for establishing national antibiotic monitoring systems include starting where the greatest impact can be anticipated in a given context and alignment with other programmes.

**Conclusions:**

The agricultural, population, epidemiological, cultural and economic contexts of different countries mean different starting points for establishing antibiotic monitoring. This project characterised an *action-oriented prioritisation* approach, targeting collection and collation of antibiotic data that have the highest likelihood of affecting change that can achieve impact. Such an approach is risk-based, prioritising monitoring of the antibiotic use that poses greatest risks as locally defined and is feasible to change; sustainable, aligning local expertise, infrastructure and technology with other country priorities; and transparent, ensuring evidence availability within the system alongside reporting ‘up and out.’ Effective national antibiotic monitoring systems require a collaborative and coordinated strategy focusing on data for action.

## Plain language summary

Antibiotics play a vital role in limiting infections in humans and animals; however, their use also drives antimicrobial resistance (AMR), which reduces their effectiveness. Therefore, it is vital to understand how and where antibiotics are used in human and animal health and food production. Monitoring antibiotic use helps to identify where prescribing can be improved and environmental discharge can be reduced, as well as promote the strengthening of pharmaceutical supply chains and improve antibiotic access.

This article draws on documentary analysis and expert consultations to review and help navigate practical tools and guidance for countries aiming to monitor antibiotic use. It covers human and veterinary medicine, terrestrial and aquatic animals and plants and tracking antibiotics in the environment. Although the primary focus is on antibiotics, the monitoring systems described could be used for other medicines. Key principles are outlined to help institutions across One Health domains to design and implement effective antibiotic monitoring.

The sheer number of antibiotics in various formulations, combined with the complexity of supply chains and limited resources for monitoring, make fully comprehensive approaches unrealistic. This paper emphasises the prioritisation of data collection that can inform action and in formats that enable meaningful analysis for use at multiple levels.

## Introduction

Antimicrobials include biologically active substances used to treat, prevent and control infections in humans, animals and plants. They have become lynchpins of our modern systems of healthcare, animal health and agriculture. Increasing concerns about the widespread impact of antimicrobial-resistant microbes, largely driven by the widespread use of antimicrobials and their discharge into the environment, have heightened calls for improved data describing antimicrobial use (AMU), where and which antimicrobials are being used and discarded within and beyond formal human health, animal health and agri-food systems, as well as environmental pollution. At the same time, ensuring access to essential, quality-assured antimicrobials remains a challenge that requires a systematic understanding of their availability across human, animal and plant health systems.

Antimicrobial resistance (AMR) is a complex and multifaceted global problem with drivers and effects across human, animal, plant and environmental domains. Projections suggest that by 2050 1.91 million deaths may be directly attributable to bacterial AMR and 8.22 million deaths may be associated with bacterial AMR annually ( Naghavi *et al.*, 2024). Furthermore, AMR could lead to a global GDP decrease of 2 to 3.5% by 2050, impacting low-income countries the hardest ( World Bank, 2017). Healthcare could see a cost surge of up to $1 trillion annually and livestock yields could substantially drop, exacerbating economic disparities ( World Bank, 2017). The spillover of resistant pathogens from livestock to humans could reduce the Global GDP by $5.2 trillion between 2025–2050 ( WOAH, 2024a).

The widespread use of antimicrobials across human health, animal health and production accelerates the emergence and spread of resistant microorganisms within human populations ( Spotts & Cervantes, 1995), across animal species and in the environment ( United Nations Environment Programme, 2023). Estimates of the global trajectory of AMU suggest an increase in the coming years in both animals (terrestrial and aquatic, henceforth in this paper referred to as ‘animals’ unless specific classification applies) ( Acosta *et al.*, 2025) and humans ( Klein *et al.*, 2024). In addition, ongoing exposure and vulnerability to infectious diseases, together with a lack of water, sanitation and hygiene provision, as well as empiric treatment, drive demand for antimicrobials. While excess use is in some cases a problem, so too is access, especially in countries constrained by supply chain weaknesses, medicines shortages, market pressures, and regulatory barriers to authorize new drugs ( Pan American Health Organization, 2021). Monitoring the use of antimicrobials in humans, animals and plants is therefore critical to provide an evidence-based approach to optimising AMU, balancing needs for access to antimicrobials that will be effective in the prevailing resistance situation with concerns of overuse and minimisng discharges to the environment. Countries have signed up to global targets on optimising AMU across One Health domains but there is growing recognition that aggregate ‘consumption’ level data is insufficient alone to guide action (the WHO now refers to ‘medicine-level’ and ‘clinical’ data as ‘antimicrobial use’ and has ceased reference to antimicrobial ‘consumption’ ( WHO, 2025)). However, knowing the ongoing scale of manufacturing, circulation, use and disposal of different antimicrobials is challenging. Nationally, AMR coordinating committees, drug regulatory authorities, procurement bodies and other entities managing supply, availability, use and disposal of antimicrobials and possible contamination of food and the environment by antimicrobials and their residues, need detailed data on their production, distribution, usage, storage and disposal to set baselines and to prioritize and track interventions. The complexity of distribution channels of antimicrobials and the systems where they are used, in human, animal and plant health and in food systems, is reflected in the numerous tools and guidelines on monitoring AMU and the lack of harmonisation in data collection ( Mesa Varona *et al.*, 2020). Given that many of the same antimicrobial molecules are used across these domains, high-level coordination of monitoring activities has been strongly encouraged ( IACG, 2018). However, despite increasing number of countries reporting AMU data in human health and animal domains ( WHO, 2025; WOAH, 2024b), the sources used may not provide sufficiently accurate, granular, or representative data to inform proposed actions nationally ( Kitutu *et al.*, 2023). Subnational data are also required to monitor programmes and understand and manage AMU, although metrics vary ( Collineau *et al.*, 2017). Interpretation of these data is challenging, with limited benchmarks to inform action and the complexity of metrics that balance access and excess in given contexts. Furthermore, current metrics are sufficiently different to make cross-sector comparison of AMU challenging. There is a need to identify key areas to prioritise to enable systematic monitoring of antimicrobials and inform subsequent actions to support countries with limited resources to monitor and respond to antimicrobial needs ( Frost *et al.*, 2021).

A system to monitor antimicrobials may comprise multiple data sources, types of institution and data platforms. It may sit within a national medicines monitoring programme or be developed for specific antimicrobials such as antibiotics. In many countries there are well-established systems for monitoring antiretrovirals, antimalarials and TB drugs, associated with disease-specific programmes for human health. Although these programmes could ultimately become a part of a wider system for antimicrobial and other medicines monitoring, this paper focuses on the supply, use and disposal of *antibiotics* across the One Health spectrum. Resistance to antifungal medicines is of growing concern, with negative impacts for human health, food production and ecosystems and given their widespread use in plants, the importance of monitoring is becoming apparent ( Fisher *et al.*, 2024). Furthermore, given the significant role that the environment plays in the development, transmission and spread of AMR, monitoring antimicrobials and AMR in the environment, along with their associated risks, is critical ( UNEP, 2023; Babu Rajendran *et al.*, 2023). This raises the need for systematic monitoring to ultimately include pesticidal and bactericidal antimicrobial agents. However, tools for the systematic monitoring of these wider antimicrobial groups and uses are even less well developed and could be informed by an effective system for antibioitc monitoring. Thus, in this paper, we focus on antibiotics as an already challenging, wide-ranging and complex group of medicines for which to establish monitoring systems. The Surveillance and Epidemiology of Drug-Resistant Infections Consortium (SEDRIC), funded by the Wellcome Trust, commissioned an Antimicrobial Medicines Surveillance Working group – to include a range of individuals from around the globe with different expertise and experience – to share insights and to synthesize guidance to help tackle the gaps in the surveillance of antimicrobial medicines throughout their lifecycle. With a focus on resource constrained settings in low- and middle-income countries, the aim was to apply technical expertise and implementation experience to support country-based prioritisation for complementing antimicrobial resistance surveillance with systems for surveillance of antibiotics, or ‘antibiotic monitoring.’

This paper is aimed at a broad audience of stakeholders, including regulatory authorities and regulator networks such as the Regulatory Agencies Global Network against AMR (RAGNA), those responsible for medicine management in a country, those responsible for the design and implementation of the National Action Plan (NAP) on AMR, drug and therapeutics committees, prescribers, farmer associations, professional associations with data and the ability to take data-informed action, for-profit organisations with a stake in health (human, animal, plant, environment), food production and antibiotic medicines sales, environmental authorities, civil society organisations and end-users of antibiotics.

## Methods

The project had three areas of focus, explored through a combination of desk-based reviews, expert consultations and a workshop. For each area, insights were informed by experience with implementation in different countries, as well as a discussion of general principles and the medicine surveillance landscape.

1. Articulating the value of a system to collect, collate, analyse and present data on antibiotics along the supply/value chain in a given country context.
2. Mapping existing guidance and toolkits that contribute to antibiotic monitoring across human, animal, plant and environmental domains at different locations in country systems.
3. Characterising approaches for prioritisation amongst options for antibiotic monitoring activities across space and time in a range of settings, with a primary focus on countries with lower resource availability in the Global South.

This paper was developed iteratively through a consultative approach, following the workflow of previous successful SEDRIC working groups. A steering group was set up to lead the project, consisting of SEDRIC representatives and three consultants. Initial stakeholder mapping involved identifying key individuals through their published work and through recommendations of others working in antimicrobial use across One Health domains. We undertook consultations on the purpose, scope and content of the project with 15 of these individuals, who also assisted in suggesting others with relevant expertise. These secondary contacts provided further suggestions, leading to invitations to a total of 71 experts, of whom 50 provided active input in the form of a reference group. Of these, 25, selected to represent sectors and regions, accepted an invitation to form a Technical Working Group. The final authorship list of 40 people located in 25 countries includes members of both of these groups, representing a range of international and regional bodies, official roles at national level as well as academia. Across the three levels of engagement, representation was secured from across human, animal, plant and environmental sectors. As there was no data collection involved, no ethical approval was sought and no consent forms were used for this project.

A search of both scientific and grey literature was conducted to identify available antibiotic monitoring and surveillance tools, with the aim of identifying the tools that could be used for data collection and collation and their usefulness for collecting data at certain locations in the supply/value chain of antibiotics. Much of the experience of monitoring, and many of the tools in use, are not in the peer reviewed literature, and so a “Snowball search strategy” was used, An initial search of peer reviewed literature, using standard search terms (“antibiotic” AND “use” OR “consumption” OR “dispens*” OR “residue” AND “surveillance” OR “monitor*” OR “report*”) and review of websites of international agencies for tools and guidance was carried out. This was complimented by information from the Reference Group members from all regions and relevant disciplines, who provided links to tools and other informants. Versions of the inventory and manuscript were reviewed and revised iteratively, and reflect the input from the broad experience of participants both in terms of geography and areas of technical specialisation. Points of consensus were agreed in meetings and in manuscript review. The Inventory of the tools identified, as of 1 May 2025, for antibiotic data capture and analysis can be found in the Supplementary File. The literature search identified two types of outputs: a) antibiotic monitoring and surveillance tools (covering data collection-oriented and residue control tools), and b) guidelines, standards, and mapping/assessment tools for monitoring antibiotics.

The Technical Working Group met at the Wellcome Trust in London from 9–11 December 2024 for a workshop to discuss the importance of data collection about antibiotics, to exchange experiences from different countries and sectors, including insights on how data are currently being collected and used and possible ways forward in strengthening data collection and use for action. Proceedings from the workshop can be found in the Supplementary File.

## Findings

### Why monitor antibiotics?

The consultations and workshop engagements helped to identify a range of benefits in capturing, collating and using data for understanding the distribution of antibiotics, their use and disposal both to inform action on AMR and for wider health system performance, human animal and plant health and food production, pharmaceutical market regulation, pharmaceutical manufacturing, pollution prevention and control and other societal reasons. Monitoring and analysing the use of antibiotics across sectors is one component of a fully integrated AMR surveillance system which also incorporates resistance patterns across sectors. A robust AMU monitoring system provides essential data to guide and evaluate interventions at multiple levels.

**Improving Access to Antibiotics**

• Support the regulation of antibiotic supply chains to improve equitable and affordable access to quality antibiotics essential for human and animal health, while preventing overuse and misuse and minimizing discharges into the environment.
• Support the development of essential medicine lists (EMLs, human and veterinary) at national and facility levels.
• Enhance functionality and reliability of medicine distribution networks.
• Inform upstream supply chain interventions, such as market shaping, pooled procurement and antibiotic pricing strategies.
• Support market regulation and sustainable procurement planning, ensuring optimal antibiotic supply and pricing and compliance with environmental measures.
• Support monitoring of stockouts, shortages and direct supply to consumers.
• Provide information and data for advocacy and decision making.
• Provide insights into potential environmental contamination by antibiotics and inform preventative and management measures and interventions, including around manufacturing.

**Improving human and animal health outcomes**

• Improve treatment outcomes and reduce unnecessary antibiotic use through preventative actions and developing and monitoring adherence to standard treatment guidelines at national and facility levels.
• Monitor the impact of interventions including those related to prevention from different sectors.

**Addressing Antimicrobial Resistance**

• Understanding the association between and significance of AMU and AMR risks and drivers within and between different sectors (human, animal, plant, environment).
• Establish a baseline to track progress with AMU optimisation against national and international targets.
• Monitor effectiveness of facility- and system-level antimicrobial stewardship (AMS) interventions and regulatory strategies, assessing guideline adherence and appropriateness of use using tools such as the WHO Access, Watch and Reserve (AWaRe) classification.
• Support evidence-based multi-sectoral policy development, strengthening national and regional antimicrobial stewardship efforts.
• Inform the prioritization and implementation of mitigation strategies at national and facility levels, helping human and animal health professionals to align prescribing with resistance patterns.
• Support benchmarking of antibiotic use and safe disposal measures to help users identify scope for improvement.

**Cost Containment**

• Reduce healthcare costs by identifying inappropriate AMU, including where higher-cost products are used unnecessarily and when medicines are used when not indicated or not the appropriate dosage.
• Provide information to inform more efficient approaches to preventing and managing infection in animals ( Adamie *et al.*, 2024; WOAH, 2024a).

**Health Security, Trade and Food Safety**

• Inform national and international procurement to minimise risks of national and international shortages and stockouts of essential antibiotics and to streamline availability of new antibiotics to treat multidrug-resistant infections.
• Provide documentary evidence on compliance with international standards, norms and reporting requirements (regional and global) and progress against targets.
• Support surveillance of antibiotic residues in food, contributing to food safety and shaping domestic and cross-border trade policies.
• Promote containment, control, or barriers for pollutants to avoid the consumption-excretion cycle that occurs when inadequately treated or untreated wastewater is used to irrigate farmland, or animal manure or human waste is used to fertilize crops. This results in functional antimicrobials entering the environment unless waste management measures to address AMR development and spread are implemented ( UNEP, 2023).

**Figure 1.**
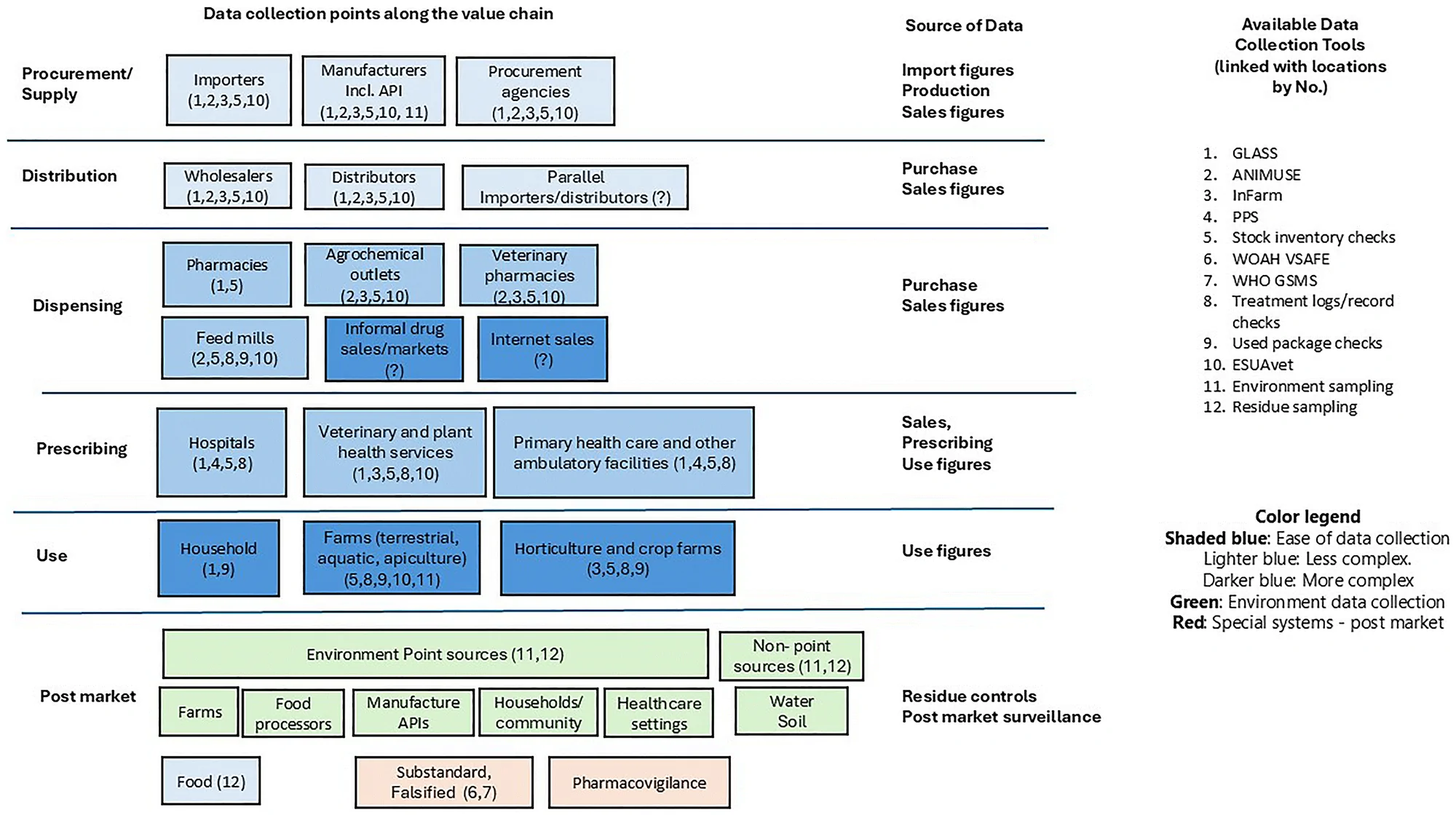
Within-country antimicrobial medicines data sources.

There was consensus, emergent through the in-person workshop and agreed by participants and subsequent contributors, that the focus for antibiotic data collection, analysis and use should be based on why the information is needed and how it would be used. Importantly, data should not be collected simply for reporting ‘out’ but should be used to inform policies, regulations and practices locally, with onward transmission of information ‘up and out’ as a secondary product of these data collection practices. Data for action may need to be more granular and targeted than data currently being collected. For example, data to design or monitor treatment interventions should be at the substance level, not just the class level. Data on total volumes used needs to be complemented by information on how and where they are being used. Box 1 provides insights from cases in Thailand, Brazil and Nigeria, where data on antibiotic use has informed action. Figure 1 shows the range of actors in different positions that can take action on the basis of AMU data.

Box 1. **Value of AMU data for action in Thailand, Brazil and Nigeria.**

**
Thailand example**

One Health stakeholders in the Health Policy and Systems Research Network contributed to the establishment of national antimicrobial consumption (AMC) surveillance in 2015. It was institutionalized for monitoring progress of NAP’s targets and indicators ( Tangcharoensathien *et al.*, 2017). The methodology was peer-reviewed by WHO and experts ensuring international comparison. It leveraged existing legal provisions requiring all manufacturers and importers to report annual volume to Thai Food and Drug Administration.

Subsequently, the system expanded to include more sectors, AMR patterns in human health and public knowledge and awareness of AMR. Since 2017, evidence has been published annually in One Health report in a publicly available dashboard ( www.ThaiAMRWatch.net), and informed the National Committee for policy decisions. The system is expected to cover antimicrobial use in hospitals and AMR-associated mortality in coming years.

Locally initiated, funded and diversified One Health AMC surveillance towards AMR related issues is not possible without capacities and sustained commitment by all One Health partners.

**Brazil example**

Since 1999, Brazil has implemented various policy actions, programs, and regulations aimed at combating AMR and ensuring proper antimicrobial management, including prohibition of antibiotic sales without prescription since 2011 ( ANVISA, 2011).

Another important action in the country was establishing the reverse logistics of medications, a component of the National Solid Waste Policy (PNRS), established by Law ( Brasil, 2010). Reverse logistics is an instrument characterized by a set of actions, procedures, and means, designed to enable the collection and return of waste to the business sector for reuse in its production cycle or environmentally appropriate final disposal. The implementation of the reverse medication policy in Brazil has shown significant results. According to data from the National Information System on Solid Waste Management (SINIR), in 2023, 448,000 kg of medications were collected at 6,294 collection points distributed across 648 Brazilian municipalities. The LogMed System, created and managed by 16 entities in the pharmaceutical sector, has been fundamental for the operationalization of reverse logistics of medications in the country. From the beginning of its implementation until May 2025, LogMed reached the milestone of more than 1,000 tons of medications collected. Currently, the system is present in 650 municipalities, located in all States of the Federation and the Federal District, with approximately 6,800 collection points installed in pharmacies distributed throughout the country.

**Nigeria example**

Nigeria has been monitoring antimicrobial agents used in animals consecutively for the past nine years through the WOAH ANIMUSE database. Annual data collected from the list of registered products from the national regulatory authority (National Agency for Food and Drug Administration and Control - NAFDAC) are analyzed and reported to WOAH and shared during meetings of the national AMR technical working group and AMR coordination committee. AMU data was used by the Federal Ministry of Livestock Development to inform the development of a national policy on AMR and use which has since been validated and awaiting endorsement by policy makers. In addition, NAFDAC used the AMU data to develop the following regulatory directives: prohibiting the use of antibiotics as growth promoters in livestock and poultry; prohibiting the use of banned veterinary pharmaceuticals in food producing animals; and ban the use of colistin as coccidiostat in animal feed. The data also informed the decision by the regulatory authority to prohibit the importation of fixed-dose combinations of antimicrobials.

***Domain specific considerations***

**Monitoring antibiotic use in plants**

Antibiotics are also used in plant production ( Taylor & Reeder, 2020), but the scale and impact is less well understood. There are limited data on volumes used, and the extent to which this results in antibiotic resistance in humans but good evidence that antifungal use in plant production accelerates resistance in humans ( Lockhart *et al*. 2023), and that antibiotic exposure decreases, the microbial biomass, diversity and enzymatic activity in soils ( Wang *et al*. 2024) and increases antibiotic resistance locally.

Antibiotics and fungicide exposure results from direct use in plant production and protection, together with the use of reclaimed wastewater for crop irrigation, use of manure as fertilizer and inadequate waste management. However, a systematic approach to monitoring is still evolving and even the terminology used varies from other sectors. Antibiotics are used very differently between countries. For example, in Europe and West Africa, the use of antibiotics for plant production and protection is generally not authorised ( Verhaegen *et al.*, 2023). In some other parts of the world, when they are used, they are described as pesticides – sometimes to treat bacterial infection, sometimes because of their effectiveness against fungi and sometimes in combination with other agents ( Stockwell & Duffy, 2012).

The main crops where antibiotics are used include citrus, rice, apples, pears, kiwi, tomatoes and potatoes. The most commonly used antibiotics are streptomycin, oxytetracycline, kasuagamycin, oxolinic acid and gentamicin although examples exist of many other antibiotics being used ( Chanvatik *et al.*, 2019; Taylor & Reeder, 2020). The risks of antibiotic resistance development in plant pathogenic bacteria are clear; however, the scale of antibiotic resistance gene transfer to animal and human bacteria and to the wider soil microbiome, is not ( Stockwell & Duffy, 2012; Verhaegen *et al.*, 2023). Moreover, farm workers may be exposed to hazardous amounts of antimicrobials if they are not applied appropriately or without the use of proper personal protective equipment ( UNEP, 2023).

Studies have identified antibiotic use in many countries for the control of plant pathogenic bacteria but there is a need for stronger data to assess whether use of antibiotics in plant production contributes to AMR in humans and animals ( Spotts & Cervantes, 1995; Taylor & Reeder, 2020, Verhaegen *et al*. 2023, 2024). Precise amounts are difficult to assess because of the lack of monitoring and may still be more substantial than first thought ( Chanvatik *et al.*, 2019; McManus, 2014). In 2023, the International Plant Protection Convention (IPPC) launched two early scoping surveys to collect data on antibiotic and fungicide use ( IPPC, 2024). Monitoring systems are still developing. FAOSTAT collects data on pesticide use, including fungicides and bactericides and a new data collection questionnaire, building on the IPPC surveys, is now being integrated into InFARM ( IPPC, 2025). This questionnaire is intended to gather information on the use of antibiotics in plants from both quantitative and qualitative perspectives. Data collected include the amounts of antibiotics used, types of antibiotics used and authorized, crops treated, targeted plant diseases, and the application practices. Collectively, these data will provide a more comprehensive understanding of how antibiotics are used in plants and the factors influencing their use.

***Environmental monitoring of antibiotics***

The monitoring of antibiotics in the environment can serve several purposes ( Huijbers *et al.*, 2019; UNEP, 2023). One is to provide a reflection of regional and national use, an approach that has been used extensively for monitoring illegal substance use in the past. In many parts of the world, reliable usage or sales data for antibiotics are lacking or difficult and expensive to generate. Environmental surveillance can have a particular value in such regions. While samples from various sources may be useful for particular investigations, including high-concentration areas such as hospitals or manufacturing runoffs, a promising approach for understanding population-wide antibiotic use is to sample untreated municipal wastewater. One wastewater sample may reflect antibiotic use by thousands or potentially even millions of people; thus, it has the potential to be highly cost-efficient for generating data on use in humans. A prerequisite for interpretation is triangulation with high-quality sales data. Recently, a Swedish study performed initial benchmarking for over 50 pharmaceuticals, including some antibiotics ( Osena *et al.*, 2025). The overall conclusion was that analyses of pharmaceuticals in wastewater could, in most cases, predict usage with precision within a factor of two. Notably, no beta-lactam antibiotics were included in this evaluation.

An additional objective of the environmental surveillance of antibiotics is to assess the risks associated with their release. For antibiotics, there are two conceptually different types of risk. One is the ecotoxicological risk: that ecosystem functions, services, or diversity are affected by antibiotic residues ( Brandt *et al.*, 2015). The other – greater – risk, given the potency of antibiotics for microbes, is to drive antimicrobial resistance. Although bacterial communities can show resilience to antimicrobial stressors, this may increase the potential for selection and co-selection of resistance as well as enabling transmission of AMR in the environment ( Hanna *et al.*, 2023; Larsson & Flach, 2022). Note that this is not to be seen as a risk to the environment as such, but rather as a threat to our future use of antibiotics as therapeutic agents ( Larsson *et al.*, 2023). The environment is an important transmission route for many bacteria, particularly faecal transmission. It also serves as a vast and diverse source of resistance genes that gain mobility and make their way into bacterial pathogens. Sources of antibiotics of value to survey include both releases from use and direct discharges from manufacturing ( WHO & UNEP, 2024). However, the specific information required to understand the significance of antibiotics in wastewater for AMR emergence and their impact on ecosystems remains an area for research.

Formalised environmental monitoring of antibiotics remains limited. The “Pharmaceuticals in the Environment” database, compiled by the German Environment Agency, analyzed 2,062 peer-reviewed publications up to 2020, focusing on maximum environmental concentrations worldwide (Umwelt Bundesamt, 2024). In 2019, the University of York, UK launched the Global Monitoring of Pharmaceuticals Project to assess the scale and drivers of pharmaceutical contamination in global freshwater systems ( University of York, 2019). A few antibiotics are monitored under the European Water Framework Directive, which requires EU member states to track their presence in water bodies ( European Parliament and the Council, 2000). However, many low-resource settings are not connected to centralised sewage systems and do not have the laboratory capacity or sufficient supplies of consumables to process the required samples. Even where such sampling and analysis is possible, for example with centralised chemical analyses, there remain limitations to the widespread use of wastewater sampling in that, while it can provide a high-level picture of circulating antibiotics, these data lack granularity. Wastewater analysis does not provide information on how or by whom the antibiotics are used and municipal systems may mix inputs from different sources, such as hospitals, industry and slaughterhouses. Because of dilution and flow variability, these data also do not indicate consumption levels. Some types of antibiotics may also be missed due to transformation and degradation. For example, the instability of many beta-lactams in the environment would create uncertainty over whether this important class of antibiotics is adequately captured.

***Data needs will vary depending on the use case***

The type and quantity of AMU data needed will depend on the action the data will inform. There are essentially two types of data to consider: medicine-based data that provide information on the type and volume of antibiotics (what, how much) and individual-based data (person, herd, animal) that provide additional information on the nature of treatment provided, the recipient and in some instances the indication.

Population-level data can demonstrate total consumption rates, which are important data for all sectors and are often linked to targets and policy objectives. Stratification of population data by the AWaRe or VetAWaRe (in the early stages of development) systems will facilitate the interpretation, communication and use of data. AWaRe provides an indication of prescribing quality and is the basis of global and national targets in human health. Population data can also indicate overuse of particular molecules, such as WATCH or not-recommended antibiotics in human health or Medically Important Antibiotics (MIA) in agriculture ( WHO, 2024a), as well as apparent underuse of other molecules that may reflect problems with access and the supply chain. Individual-based data are required to understand how and where antibiotics are actually used to inform and monitor treatment guidelines and behavioural interventions.

### Mapping existing systems, tools, methods, guidance and resources for antibiotic monitoring systems

***Global systems for monitoring antibiotic use***

At the global level, the Quadripartite organisations (FAO, UNEP, WHO and WOAH) have developed tools to collect, categorise and analyse antibiotic sales and use data. These are overarching tools with multiple functions that provide platforms for sharing and standardising data, highlighting the importance of triangulation for data quality and accuracy.

The Global Antimicrobial Resistance and Use Surveillance System (GLASS) AMU by the WHO is a platform that displays country-specific information based on the ATC system for classifying antibiotics and DDD for expressing volume. It includes data sources, population coverage, level of care (community vs. hospital), healthcare sector (public vs. private) and data type (import, distribution, dispensing, or mixed) while also recording any medicine shortages during the reporting period. There is also a Point prevalence survey tool collecting data on hospital use, and guidance on the analysis and use of AMU data. WHO regions have developed guidance on developing AMU surveillance in humans at a national level ( WHO Regional Office for the Western Pacific, 2026).

ANIMUSE is a global online database that allows countries to report, access, analyse and communicate data on antibiotics intended for use in animals to different stakeholders, developed and managed by WOAH. It assists countries in collecting standardised data on imports and sales and provides an estimation of the use of antibiotics in animals at the country level. There are several ways to report the data, from qualitative information to detailed quantitative data on the active ingredients, species groups and administration routes. ANIMUSE provides calculation tools to standardise AMU data and adjust for estimated animal biomass (active ingredient in mg/kg animal biomass), with an integrated function to receive digitised data from national authorities. ANIMUSE provides valuable data at a national level based primarily on sales and import figures. Complementing this with additional insights on actual use at the farm level could enhance understanding of usage patterns and inform more locally targeted interventions.

In parallel with ANIMUSE’s official submissions from the WOAH focal points at the country level, WOAH gathers information in the form of an inventory, information on projects and studies monitoring antimicrobial use (AMU) at the *field level* across various regions and animal species from a wide range of sources, veterinary services, academia, NGOs, research institutions and others. The submissions are not endorsed by WOAH, but the inventory is a good source for learning about projects on AMU at the farm level around the globe.

InFARM is a global information system consisting of an IT platform and related FAO activities that assist countries in collecting, collating, analysing, visualizing and effectively utilizing their AMR monitoring and surveillance data primarily from livestock and aquaculture, along with their associated food products. In addition to its initial focus, FAO is currently expanding the scope of evidence-generation activities to other areas under the remit of the Organization, such as AMR and AMU monitoring in the environment in close proximity to food production, monitoring of the use of antibiotics in plants and monitoring of antibiotic residues in food.

UNEP aims to establish a science- and risk-based system to analyse antimicrobial residues and resistance in the environment, including identifying appropriate methods for environmental surveillance. The system is to be complementary to existing global surveillance systems.

These tools can incorporate data from locations along the supply chain, although ANIMUSE, for instance, mainly obtains data from imports, manufacturing and sales. Approaches to data collection become more complex, more numerous and less standardised with increasing distance from import or manufacturing points, to, for example, at the household or farm level. These include variously designed questionnaires, face-to-face interviews, stock inventory checks, treatment logs/records and used packaging of antibiotics ( Queenan *et al.*, 2017). In low-resource settings, effective systems for monitoring use need to integrate these diverse data sources while addressing persistent challenges in data capture, standardisation and data reliability at the end-user level ( Schar *et al.*, 2018).

***Regional, national and commercially run systems***

Other well-structured antibiotic medicine data collection models include the European ESUAvet system, which replaced the European Surveillance of Veterinary Antimicrobial Consumption (ESVAC) system that ran from 2009 to 2023. ESUAvet collects and analyses data on sales volumes of veterinary antimicrobials and their use in animals in the European Union (EU). The European Surveillance of Antimicrobial Consumption Network (ESAC-Net) by the European Centre for Disease Prevention and Control (ECDC) tracks and analyses human AMU in the European Union and European Economic Area countries using data from both community and hospital settings. The outcome of the surveillance is analysed jointly by the EMA, ECDC and EFSA to better understand the potential relationship between the consumption of antimicrobials by humans and animals and the occurrence of antimicrobial resistance. The results are published in the Joint Inter-Agency Antimicrobial Consumption and Resistance Analysis (JIACRA) reports ( ECDC, EFSA & EMA, 2024). The Western Pacific Antimicrobial Consumption Surveillance system WPRACCS is a system designed to support Member States of the WHO Western Pacific Region to capture data on antimicrobial consumption (AMC) at national, hospital and community pharmacy levels. Thailand, for example, has made notable progress by producing a One Health report that integrates data on AMU and AMR in both human and animal sectors ( Lekagul *et al.*, 2023). In Brazil the National System for the Management of Controlled Products (SNGPC) was implemented in 2007 ( ANVISA, 2014). It is a health information system for data on the production, manipulation, distribution, prescription, dispensation and consumption of medicines and pharmaceutical ingredients by pharmacies and drugstores.

Sales data in many countries are systematically collected and analysed by commercial organisations such as IQVIA ( IQVIA, 2024). Accessing these commercial data can however be costly. Some limitations to the IQVIA system are related to sampling risk as IQVIA relies on national sample surveys conducted across the pharmaceutical sales distribution chain. IQVIA data predominantly reflect sales from private pharmacies and wholesalers, potentially leading to an underestimation of antibiotic consumption by public healthcare services. Commercial data derived from sampled sources can yield conclusions that differ from those based on administrative datasets that encompass the entire population. In many countries, routinely collected administrative data and robust information systems are lacking, making IQVIA data a major source of information. Where other routine data systems exist, triangulation between sources is a useful way to validate and understand systems ( Marin *et al.*, 2025).

***Locating and generating antibiotic data***

To understand antibiotic movements within a country and identify locations where data may be available, the pharmaceutical supply/value chain was mapped from production, through intermediaries to the final user. It was adapted to visualize the various potential antibiotic data sources and collection points across sectors (human, animal, plant and environment), elucidate what data are potentially available where (public, private, quasi-government, informal) and how data collection can be organized. Figure 1 illustrates possible locations of antibiotic data.

Collecting data on antibiotics at different collection points in a supply chain is a challenging task, as it requires collaboration between various stakeholders, public, faith-based and private institutions. It may also require capturing information about the cross-border circulation of antibiotics, which may involve additional authorities.

At the national level, collecting data across the levels of the supply chain is often facilitated by the presence and implementation of regulations requiring data submission to authorities. At the local level, data can be shared through local initiatives, often on a voluntary basis.

Sometimes, the benefits of collecting and sharing data are not readily visible to the entity providing the information, which comes at a cost to labour as well as commercial interests. Lack of communication between stakeholders and lack of knowledge at the national level of what data are collected by various stakeholders, including private entities, the public administration and sometimes specific research projects funded by academic institutions and philanthropic institutions, make it more difficult to develop a coherent, consistent and repeatable approach to monitoring antibiotics and can lead to multiplication and/or obscuring of efforts (see Box 2). Public entities may be asked to report to public, academic and even private institutions simultaneously, all with different variables. This implies an economic burden, especially for public entities ( Hegewisch-Taylor *et al.*, 2022).

Box 2. **Antibiotic use data collection in fragmented health systems; the case of Mexico.**

Antibiotic monitoring in Mexico has been inconsistently implemented. The challenge is not the absence of data collection initiatives, but rather the need to align and integrate the many efforts across a fragmented health system where multiple public sub-systems coexist with a large and diverse private sector. This results in fragmented and incomplete antibiotic use (ABU) data that often fail to contribute to a government-led surveillance system and decision-making.

For instance, the public health sector alone employs over 60 distinct Electronic Health Record (EHR) systems, with many services lacking EHR entirely ( Neme-Meunier, 2019). Although these systems collect prescription data at the institutional level, the lack of standardization and interoperability limits their utility for analysis at the national level. Additionally, the extraction of ABU data from dispensing records in public hospitals is frequently impeded by insufficient allocation of human resources and limited training, which is essential for generating ATC/DDD and AWaRE information. Despite the availability of electronic databases for certain public institutions ( Lopes *et al.*, 2022) ABU data are rarely communicated publicly to inform policy or practice, whether in hospitals or outpatient settings. In the private sector, while some high-complexity hospitals conduct antibiotic monitoring, they are not required to report their data to the government. No ABU information is collected in the private outpatient sector, although some national studies have been conducted using data from IQVIA ( Santa-Ana-Tellez, 2013).

Of note, much of the available ABU data in Mexico originates from academic collaborations with hospitals. The university network PUCRA monitors antibiotic use in 47 hospitals, reporting its ATC/DDD results annually. Additionally, other academic groups have conducted and published AMU surveys (PPS). However, none of these AMU datasets are integrated into government systems or used to inform decision-making. For example, these studies consistently document excessive use of third-generation cephalosporins (3GCs), alongside increasing resistance to 3GCs in hospital pathogens. This evidence has not been used to design or evaluate nationwide interventions, which underlines the need to strengthen collaboration on AMU between public health authorities and other entities.

When it comes to collecting data at different levels of the supply chain, triangulation of data is highlighted in the global guidance documents to avoid duplication and identify gaps in the monitoring system.

As antibiotics move through the system from manufacturing towards the end user and their disposal, the complexity of collecting data from multiple sources can increase, but so too does the granularity of information. Figure 1 provides an overview of the locations and specific tools that could be used to collect or collate data at various levels in the supply chain. A list of available tools for collecting, managing, analysing and disseminating antibiotic medicines data is available in tables in the Inventory ( Supplementary File). While not exhaustive, this list provides a general overview of the identified tools, recognizing that not all may be suitable for every setting. The identified tools were linked to the data location (See Figure 1) and divided into categories according to their main function.

One of the tools listed (see Supplementary File, Table 2) is a global repository provided jointly by the World Veterinary Association (WVA) and WOAH, the WVA/WOAH Global Repository of Guidelines for the Responsible Use of Antimicrobials in Animal Health (WVA and WOAH, not dated).

Procurement/Supply: At the national level, the total volumes of antibiotics arriving in the country can be tracked through import and manufacturing totals. Data on imports may be collected from customs. Import/purchase figures may be sourced from licensed import companies and central procurement bodies such as the central medical stores and the amounts of antibiotics manufactured in-country may be accessible to the Drug Regulatory Authorities. Specific tools to collect and receive data at that level are the global-level tools GLASS AMU and ANIMUSE.

Customs data can be challenging, as customs codes do not necessarily reflect the active ingredients or distinguish antibiotics from other pharmaceuticals. In addition, for veterinary antibiotics the Customs data would not provide clear information on which species the product will be used for. Illicit flows will not be captured. As a result, customs data alone are rarely sufficiently accurate for monitoring antibiotics. Effective collaboration between customs authorities and agencies responsible for AMU data collection is required to obtain useful data.

Obtaining data from pharmaceutical manufacturers builds, in some countries and regions, on legal backing and incentives for manufacturers to report production and sales figures ( European Medicines Agency, 2023; World Organisation for Animal Health, 2022).

Countries with robust legislation on pharmaceuticals would typically link the reporting obligation of market authorisation holders to the licensing procedures ( European Medicines Agency, 2023; Sumpradit *et al.*, 2017; Sumpradit *et al.*, 2021; South Africa Department of Health, 2018).

Methods for collecting data from manufacturers and market authorisation holders include mandatory periodic reporting of national sales and exports cross-checking it with records on production and market authorisation. If there are no systems for reporting, periodic audits and inventory checks could also provide an idea of stocks. More advanced systems could include barcodes and batch-tracking systems. Moreover, commercial sensitivity and limited enforcement capacity can hinder compliance, especially in low-resource settings.

In some countries, pharmaceutical donation programmes, or medicines for specific programmes are a substantial source of medicines, which should be included in national totals.

Several countries have been developing electronic single-window systems aimed at improving coordination and sharing of data amongst trade regulatory agencies and one-stop border posts to facilitate and standardise trade, where border controls include customs, health, animal health and phytosanitary authorities. Countries implementing such systems include Rwanda, Uganda and Kenya. Another possible source of data could be the systems in place for import licensing or registration.

Random unannounced inventory checks/audits can provide an indication of which drugs are being supplied but cannot provide an indication of total quantities. Tracking the import and manufacture of Active Pharmaceutical Ingredients (API) may be outside the scope of drug regulatory authorities. It would be possible to account for APIs through the sale of antibiotics produced from them unless the APIs are used for compounding, where they might fall outside any monitoring.

Distribution: Wholesalers and distributors can provide sales data disaggregated by antimicrobial class and client type. This could provide valuable information on the distribution of antibiotics within and outside the country and possible hotspots where unexpected volumes or classes of antimicrobials are identified. GLASS and ANIMUSE are designed to receive this data and random inventory checks can also be used to identify what is being used, particularly if there are no digital records. In practice, some of the challenges in collecting robust data on the distribution level include fragmented data systems, where distributors have different systems for data on purchases and sales. The unwillingness of private companies to provide data might also hamper antibiotic monitoring and unclear and/or overlapping mandates of national authorities can lead to gaps in coverage. The sheer number of different products in different formulations makes collating information and analysing it challenging. In some countries there may be hundreds of different brands and formulations of the same molecule. Digitisation – and standard approaches to analysis – could facilitate data processing.

Dispensing: This situation becomes more complex as the number of players increases. In pharmacies, veterinary clinics/pharmacies, agro-shops and feed mills it could be possible to access prescription data in addition to purchase and sales figures, gaining information on the prescribers and the end user. In feed mills, data on the purchase of premixes containing antibiotics would be a specific form of data and sales of medicated feed. Informal drug outlets are a specific challenge at this level of the value/supply chain. Checks on treatment and prescription logs and records could add to the granularity of the data obtained. Challenges to overcome in data collection at this level include the lack of digitised systems, imperfect recording and over-the-counter sales of antibiotics, which are common in many countries, especially in those with limited resources. Collecting data manually during audits/inventory checks is resource-demanding and the sites to collect data would have to be selected carefully to ensure that they are representative of the wider population. Some ways to improve data collection at this level would be to mandate the use of a standardised format for record keeping according to the requirements of the regulatory authority to facilitate obtaining data in a standardised format with required variables and report according to the agreed schedule or provide incentives to the dispensers to submit standardised data periodically. Possible solutions to overcome challenges at this level could be to link reporting to licensing with regulatory enforcement according to policies around the use of antibiotics, train dispensers in stewardship and reporting and liaise with non-governmental organisations to carry out periodic surveys. One approach worth exploring is to introduce public-private partnerships (PPPs) with pharmacy organisations, pharmacy chains, or with companies that develop and provide electronic medical records and billing systems to provide standardised data, assist in data collection and develop electronic data collection systems and IT platforms to reduce the labour costs of collecting data. When electronic systems exist and reporting is mandatory, as in Brazil for all human and veterinary prescriptions, this can provide valuable information on use and the supply chain (See Supplementary File: Annex). Some countries are developing and introducing digital dispensing logs with user-friendly mobile apps. The purpose being to establish a system that is less time-consuming and provides standardised, easily accessible data on use that can be compiled and analysed. The WPRACSS community module offers a tool for pharmacies and drug vendors to collect data on dispensed antimicrobials by means of a mobile application that visualizes the antimicrobials most commonly dispensed by the WHO AWaRe classification ( https://play.google.com/store/apps/details?id=org.who.WPRACSS&pli=1). These results can be used to inform discussions on dispensing practices and follow-up antimicrobial stewardship interventions at the local and national levels. Internet sales are growing in many countries. This parallel supply of medicines may be seen as a further complication but could be an opportunity if appropriate systems for data capture and monitoring are established.

Prescriber: This level is complex, with public, private and faith-based health care facilities, veterinary clinics, other actors in animal health such as non-governmental organisations/charities; plant health services, and often paraprofessionals who may also prescribe antibiotics. In some contexts, community health workers prescribe antibiotics for a variety of specific uses. Robust procedures need to be established by the competent authorities to protect personal data, underpinning data-sharing agreements between parties. In general, obtaining data from the public sector is easier, as others may have commercial and compliance concerns or may require incentives to collect and report on data. Antibiotic flow between the human and animal sectors is often reported, making the capture of a whole-of-market picture more important.

Monitoring AMU in hospitals is necessary given the high levels of use, particularly of Watch and Reserve and antibiotics, the risks of AMR and because this is often where stewardship programmes start. Countries can choose between two globally recognised tools for undertaking hospital point prevalence surveys (PPS): the Global Point Prevalence Survey (Global-PPS) and the WHO methodology. The Global PPS is a web-based tool that supports the collection and analysis of data from inpatients and outpatients of all clinical specialties and age groups and provides tailored feedback and recommendations, thus supporting clinical stewardship programmes and research. The WHO PPS, a component of GLASS, collects more demographic data and has a greater public health and policy focus. If the objective is to monitor hospital use as a component of the national system, then GLASS PPS is appropriate, although some facilities are deterred by the level of detail required by this tool. Other standard tools include the Point Prevalence Survey of Healthcare-Associated Infections (HAIs) and Antimicrobial Use in European Acute Care Hospitals. The PPS for primary care is currently under development. In addition to structured PPS tools, stock inventory checks, procurement data and treatment records can be collected. The percentage of all patients attending primary care clinics receiving an antibiotic was a standard indicator in rational use of medicine programmes ( WHO, 1993), but this is now more challenging to interpret and benchmark as the case mix in primary care changes. It should be noted that the global tools can be adapted to serve regional or national priorities and settings. For example, the Pan American Health Organization (PAHO) has adapted WHO tools and trained hospitals to conduct PPS in Latin American Countries ( Levy Hara *et al.*, 2022). Where veterinary hospitals exist, similar AMU monitoring tools could be relevant.

Some of the challenges encountered when considering the collection of data at the prescriber level are the lack of incentives and the representativeness and standardisation of the data. Lack of digitisation would increase the work related to data collection and a lack of regulation and legal requirements could also hamper data collection. The importance of using standardised reporting tools should be emphasised. With an increasing number of data locations, variations in reporting tools will likely increase.

End-user : Collecting data on actual use, including self-medication, is hampered by the vast number of locations to collect data, non-standard antibitoic formulations, which can make discriminating antibiotics from other medicines almost impossible, and reporting biases. If data are to be collected at the household or farm level, ensuring representative sampling maximises the utility of the data and the information generated.

While collecting data on total use from facilities is more resource-intensive, as there may be more data locations and different types of data management software systems in these facilities, information at this level from surveys or representative sampling will provide useful information on how antibiotics are being used, whether access and use is equitable and the impact of interventions to improve antibiotic use and disposal. The unit of analysis transitions from aggregate levels at population to treated individuals or animals. The microdata at the patient level include patient socio-demographic characteristics, clinical indication and relevant characteristics, level of health care and health sector. Animal level microdata include animal species (for both aquatic and terrestrial species), type of production system, class of antimicrobial and active ingredients, quantity used, route of administration and animal biomass and can cover companion animals, food-producing animals and working animals.

In the animal sector, there has been a lack of standardised methods for collecting data at this level of the supply chain. However, FAO and WOAH jointly developed in 2023 guidelines for monitoring antimicrobial use at the farm level. The guidelines were developed by the regional offices for Asia of FAO and WOAH and are referred to as the Regional Guidelines for the Monitoring and Surveillance of Antimicrobial Resistance, Use and Residues in Food and Agriculture (see Supplementary File Table 2) These guidelines suggest a stepwise approach including PPS Studies and prioritised sentinel sites building on national priorities/prominent animal sectors in the country, expanding to other species and production systems as the robustness of the system increases. They include examples of national systems in which data collection at the farm level has advanced.

Antibiotic use data sources at farm level are treatment logs, purchase records, questionnaires and used package collections.

There have been numerous ad hoc surveys on antibiotic use in the human sector, but systematic monitoring is challenging. Antibiotic use for pneumonia in children was tracked through the Development and Health Surveys (DHS); however, the validity of the results is compromised by diagnostic uncertainty and recall bias. Tracking whether people can access antibiotics, which are available and affordable, is important for monitoring access, but this is not yet being done. Simple crowdsourced surveys - possibly involving medical students or other interested groups would be a simple and affordable way to generate this information.

Currently, there are no standardised tools for assessing use of antibiotics in plants at farm level.

Post marketing/post use: Data on environmental contamination can be obtained by analysing antibiotic residues at sentinel sites in the vicinity of points of expected use or manufacturing, such as pharmaceutical/active pharmaceutical ingredient plants, municipal sewage systems, food processing plants, hospital waste and runoffs from farms and surface and groundwater bodies. Some countries have a system to collect data on the volume of expired antibiotics, subtracting it from the data collected along the supply chain to get a better idea of what is actually used by the population ( Aedo *et al.*, 2023). High volumes of expired drugs can be an indication of supply chain problems. It is a frequent issue, when close-to-expiry drugs are supplied through donation programmes and then have to be either rapidly prescribed or safely disposed of ( Kamba *et al.*, 2017; Pinheiro, 2008).

Many countries have a targeted residue monitoring and control programme in place as a part of their overall food safety systems. These systems can possibly identify the types of antibiotics used at a specific site of primary foodstuff production. Likewise, residue monitoring at sentinel sites in the environment, such as downstream of aquaculture farms, in municipal sewage systems, or runoff from farms, can give an indication of the type of antibiotics used in a specific setting. Non-compliance with the national or international standards should trigger further investigation but would have to be evaluated carefully to avoid misinterpretation and properly inform priority-setting and decision-making processes.

Several tools are available for collecting and testing residues in the environment and in food. Tools range from sophisticated residue-testing protocols requiring access to advanced laboratories and laboratory equipment to commercial kits for onsite testing.

Residue monitoring in food and control plans are developed to ensure food safety and detect inappropriate and possibly illegal use of antibiotics. However, excessive use of antibiotics can go undetected if withdrawal times are followed.

Drug Quality and Pharmacovigilance: Systems to monitor the quantities of medicine used should be developed in parallel with and complimentary to systems for pharmacovigilance and the Substandard and Fake medicines reporting systems, the Global Surveillance and Monitoring System (GSMS) developed by WHO and the Veterinary Surveillance System for Substandard and Falsified Veterinary Products (VSAFE) developed by WOAH. These are alarm systems, which could give an indication of circulation of SF medicines in a country or a region and trigger further investigation.

***Analysis and sharing of antibiotic monitoring data***

Several tools, guidance documents and platforms can support different components of antibiotic monitoring systems (listed in the Inventory, Supplementary File), which may be classified based on their utility: providing guidance on data formats, collecting data, categorisation and analysis of data, sharing/standardising/benchmarking and for data use/feedback loops. Some of the tools identified cover more than one of these purposes. Data sharing can be hampered by several factors, such as special agreements or restrictions on sharing, especially in the private sector. It is important to respect any confidentiality requirements from owners of the data and review limitations to sharing data in those settings, as the data could be linked to individuals or companies. One way to address confidentiality issues is to formalise data sharing and handling agreements.

***Data formats***

The format and standardisation of the data are critical as they can enhance quality and provide a platform for sharing and comparing data over time as well as across geographies and sectors. Standardised data formats have been established for human and animal health.

ATC is the global standard for monitoring drug utilization and research in humans. Medicines are classified based on their anatomical, therapeutic, pharmacological and chemical properties. The DDD is a dose unit for consistent measurements. This system enables the comparison of how different drugs are used across regions, countries and over time. It is used to track drug utilization trends, compare international patterns, assess the impact of interventions and regulations, evaluate therapy intensity and monitor shifts in prescribing. However, as DDD is the assumed average maintenance dose of a drug used for the main indication in adults, it does not reflect the actual dose to be used in individual patient treatment, limiting their use in clinical decision-making and in estimating use in the paediatric population. An alternative approach, that is also applicable to children is to measure DOT Days of therapy. However, this requires more sophisticated record-keeping and is mainly used for inpatients in well-resourced settings.

ATCVet is a system for the classification of veterinary medicines and is based on the same overall principles as the ATC system for substances used in human medicine. In many cases, veterinary antimicrobial products can be classified using the ATCvet system, created by placing the letter Q in front of the ATC code. In some cases, however, specific ATCvet codes are created, e.g. antibacterials for intramammary use (QJ51) and immunologicals (QI).

The use of a denominator in antibiotic monitoring is essential for the meaningful interpretation and comparison of data, accounting for the population at risk. Data on animal populations (terrestrial and aquatic) from the World Animal Health Information System (WAHIS) of WOAH and FAOSTAT of the FAO are used to estimate the animal biomass used as the denominator in the ANIMUSE database. The complexity of collecting AMU data in the animal sector is highlighted by a vast array of metrics and indicators used for measuring and reporting AMU in animals. These metrics can be grouped into three broad categories: count-based, weight-based and dose-based. Within each main category, different approaches to measurements and calculations, along with differences in the production systems for various animal types, result in multiple metrics and indicators, each with their own advantages and disadvantages. Synthesis Agri-Food Network (2021) discusses and provides valuable examples of the use of various metrics in their Backgrounder: Metrics and Indicators for Measuring and Reporting on Antimicrobial Use in Animals.

There is ongoing work on further develop methods such as refining the Livestock Biomass Conversion Index ( Acosta *et al.*, 2025). A widely used WOAH Animal Biomass indicator (mg/kg) considers the variability of animal weights between countries by basing it on the reported slaughtered weight. In a US study comparing different methodologies, the WOAH indicator was found to be the best method for biomass estimation in global monitoring of antimicrobial sales for use in food animals ( Bulut *et al.*, 2022).

In human health, where data may cover the entire population of a country or region, standard population estimates apply, the most preferred being DDD per 1,000 people per day. For facility-based studies, information from the health management information system (HMIS), pharmacy management information system and logistics management information system have been used to calculate denominators, such as patient admissions or bed days.

For comparisons between sectors, it is necessary to use comparable denominators. The EU, in its JIACRA reports, uses weight-based indicators (mg/kg biomass) to compare AMU in humans and animals. The European Medicines Agency has further refined its Population Correction Unit (PCU) and uses mg/PCU for animals in the ESUAvet system ( EMA, 2025).

A study carried out in Indonesia, comparing different methods, found that in medium-scale broiler farms, where resources are scarce and there is no professional oversight, a count-based method was the most suitable, highlighting the importance of carefully selecting the use indicators when considering appropriate AMU monitoring methods ( Anwar Sani *et al.*, 2023).

The above examples demonstrate the complexity of calculating the denominator and the importance of selecting the metrics that best fit the setting being studied.

***Data categorisation and analysis***

The AWaRe classification was introduced by the WHO in 2017 to standardise antibiotic categorisation, improve the procurement and management of antibiotics and help policymakers and healthcare providers understand and improve antibiotic use.

Based on the WHO Essential Medicines list, antibiotics have been classified as:

• Access: essential first-line medicines that should be widely available at all levels of care
• Watch – those required for specific indications, but for which there is concern about safety or resistance potential; and
• Reserve – treatments of last resort for multidrug resistance infections.
• Not Recommended (eg fixed dose combinations without an evidence base)

This classification underpins the global target of 70% of all antibiotics for human use as access medications ( UNGA, 2024). Access antibiotics are generally narrow spectrum, have a better safety profile and are less costly than Watch and Reserve medicines. A VetAWaRe is being developed along similar lines.

Additional tools to support analysis of use in non-human sectors are the WHO list of Medically Important Antimicrobials in Human Medicine (MIA list - formally referred to as Critically Important Antibiotics CIA) and the WOAH List of Antimicrobial Agents of Veterinary Importance (WOAH list). These are lists that indicate which molecules are of particular public and animal health interest, respectively and should be utilised to inform the development of national guidelines for responsible use in animals, inform surveillance programmes and risk analysis. The AMU data collected can be segmented into categories allowing countries to analyse trends in use of highest-priority MIAs, shifts towards lower-risk antimicrobials and sector-specific usage patterns.

***Platforms for sharing/standardising/benchmarking***

Platforms for sharing and standardising and/or benchmarking/comparison include the global platforms GLASS-AMU and ANIMUSE. The European Medicines Agency has developed the ESUAvet platform, the European Sales and Use of Antimicrobials for Veterinary Medicine which is both a collecting tool and a tool that feeds into standardising and benchmarking with annual reports on the use and trends in Europe that also feeds into the JIACRA reports.

At a national level, platforms to collect analyse and benchmark data are useful, but confidentiality issues may arise. Anonymising data and feeding data back to practitioners directly, benchmarked against their peers can be an effective way to effect behaviour change ( El Feghaly *et al.*, 2023).

***Data use/feedback loops***

The global platforms aim to serve as tools for reporting on their use to inform policies and actions. The use of platforms for informing action is being prioritised with advancing national reporting tools within global platforms, such as WOAH’s ANIMUSE and WHO’s GLASS-AMU.

Reporting at the national or local level and other communication could be technical reports for specific identified audiences. Strengthening the reporting can be achieved through other tools such as national dashboards, visualising the data to a broader audience, promoting capacity building and national actions.

Some global tools evaluate the overall systems in countries and can indirectly feed into the usage of data and feedback loops by identifying gaps in the system. Tools in this category are the FAO Progressive Management Pathway and WOAH PVS Pathway (Performance of Veterinary Services) for animal health and veterinary services. The PVS Pathway can provide tailored support to countries’ needs by targeting specific priority subjects at their request, including AMR. Another tool that can also identify gaps in the systems auditing them towards international Health Regulations is the Joint External Evaluation (JEE).

Tracking the AMR Country Self-Assessment Survey (TrACSS) also monitors the implementation of all elements of the Global Action Plan on AMR and could help identify monitoring priorities. Another important tool is the Quadripartite One Health Legislative Assessment Tool for Antimicrobial Resistance, which assists countries in reviewing their legislation related to AMR. Gaps and challenges identified in antibiotic surveillance tools and some suggested solutions per sector are listed in Table 1.

**Table 1.** Gaps and challenges in antibiotic monitoring tools and guidance per sector, with suggested solutions.

	Humans	Animal health	Environment	Plants
**Gaps**	Tools for primary care and pharmacies Standards for data collection and analysis from digital systems	Specific tools for use data collection at farm level	Standard Tools for collection and analysis of data, including benchmarking against high quality sales data.	Standard Tools for collection and analysis of data.
**Challenges**	Collecting data from informal sector and from primary care Lack of standards and benchmarks Translating complex data into useable information Lack of tools to support analysis and feedback Time and skills required to collect and analyse data Costs of sustaining a monitoring system	Collecting data at farm level. Collecting data from multiple formal/informal sources Collecting data on medicated feed. Time and skills required to collect and analyse data Costs of sustaining a monitoring system	High operational costs and limited capacity for laboratory equipment, pers. Lack of benchmarking against sales/use data Ensuring compliance with regulations to residue limits especially in informal agricultural sectors. Time and skills required to collect and analyse data Costs of sustaining a monitoring system	Access to and engagement with Agricultural sector Non-standard terminology and classification Lack of incentives Costs of sustaining a monitoring system
	Regulation on mandatory provision of data on antibiotic sales by wholesalers Primary care PPS (under development) Use sentinel sites for primary care and pharmacies Integrate AWaRe classification into the analysis and feedback of data, and link to WHO AWaRe antibiotic book to aid interpretation and move towards benchmarking Develop standards and tools for extraction and analysis of data from e-procurement systems. Invest in electronic data collection tools	Engagement with importers, distributors and retailers to provide data. Strengthen regulation regarding handling and use of antibiotics Provide incentives. Strengthen AMR surveillance in aquatics. Agreements with large commercial farms to provide data Use sentinel sites to monitor use on farms for key value chains. Invest in electronic data collection tools	Strengthen regional cooperation and sharing of laboratory facilities, establishing regional laboratories, carrying out analysis of samples from countries in the region	Raise awareness with plant producers of risks of AMU use, regulation where it exists, alternatives to antibiotics and need for monitoring. Define clear frameworks for the antibiotics allowed/ Prescribed use in plants

### Steps to prioritisation

**Figure 2.**
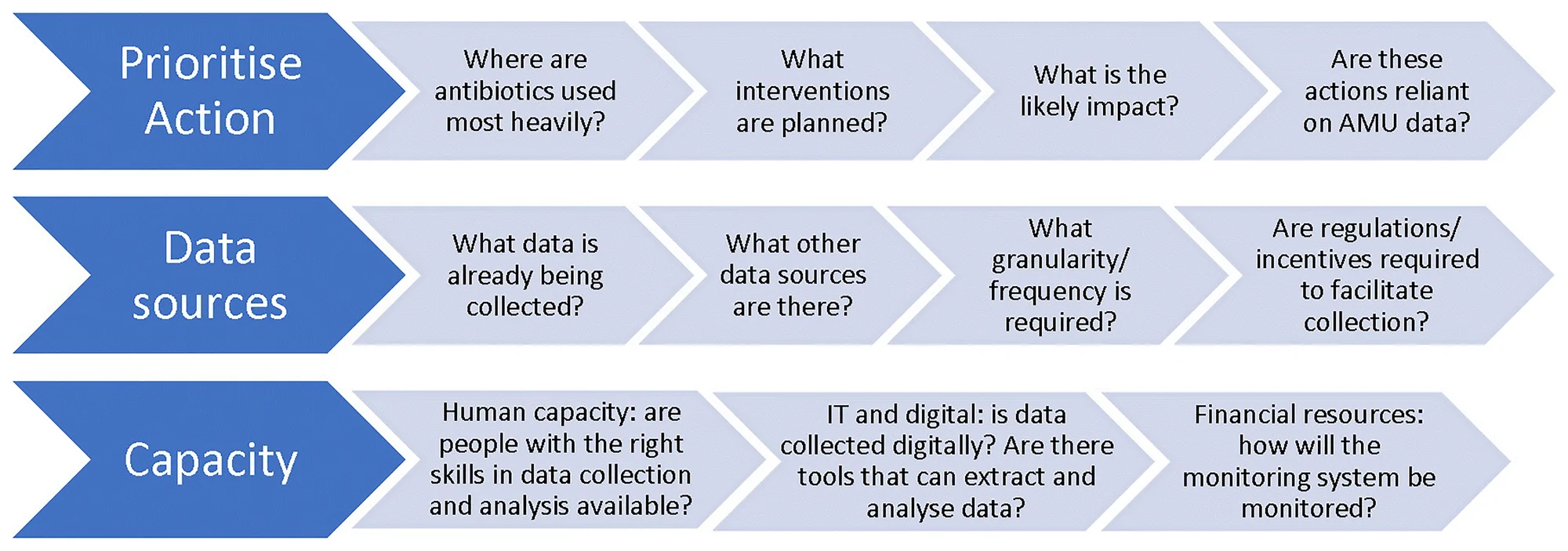
Action-oriented prioritisation steps.

The Quadripartite describe a fully integrated surveillance system as *“Continuous, collaborative, coordinated, systematic collection, collation, analysis, interpretation, communication and sharing of data on AMR and AMU and associated metadata. These includes data from humans, animals and products thereof, plants/crops and products thereof, and the environment to provide harmonized information that can be used as a basis for decisions and actions to reduce the burden of AMR and preserve the effectiveness of antimicrobial agents.”* (WHO, FAO, UNEP and WOAH, 2025). There was consensus that while a useful vision, a comprehensive approach such as this was currently beyond reach in many settings. There was also agreement that the variety of health, agricultural and manufacturing scenarios in different countries mean that the essential components of an antibiotic monitoring system will also differ. A One Health approach is important overall, with established points of connection and alignment between sectors, especially in data analysis and use; however, most actions are sector-specific. The group identified a set of steps to prioritisation that could help countries identify the most impactful areas of antibiotic monitoring systems to invest in ( Figure 2).

***Understanding the landscape***

**Figure 3.**
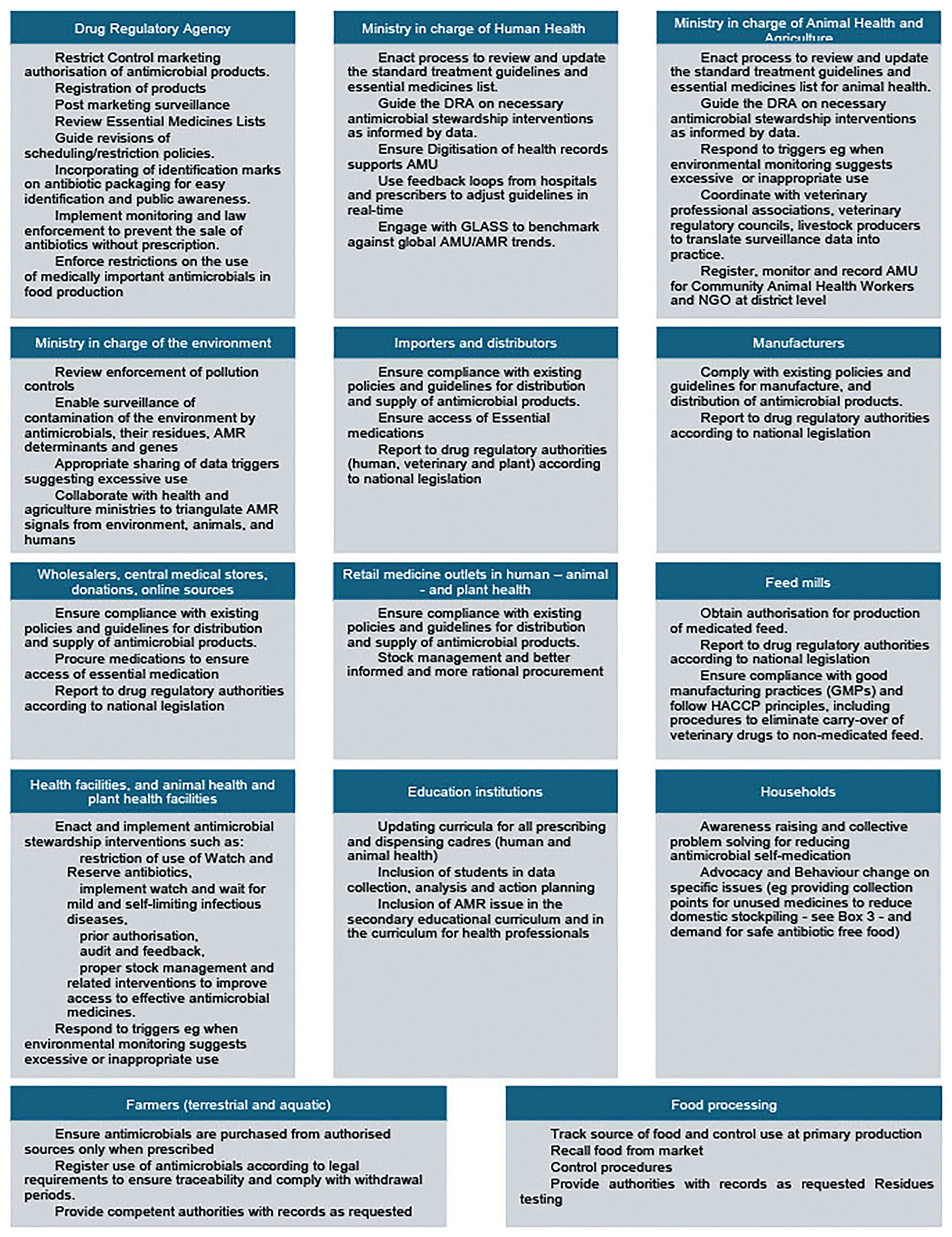
Target data users: stakeholder organisations with ability to take action on findings of antibiotic monitoring.

A key point of consensus from the working group was the prioritization of data that could inform action. Monitoring antibiotic use is a core element of monitoring NAPs for AMR and the impact of programmes designed to prevent infection and optimise use ( World Health Organization, 2015). A landscape analysis of existing sources is important to understand how and where antibiotics are used, to focus actions on the areas of greatest risk, or where gains are most likely. Figure 3 summarised the antibiotic data-informed actions that can be taken by different stakeholders.

In animal health, this means understanding which are the major value chains in livestock production and aquaculture, how and which antibiotics are currently used and how amenable this would be to change with preventative actions to reduce the use and minimize the discharge into the environment. The need to comply with export regulations can be an important incentive to encourage monitoring of use.

In the human health system, it is important to understand where most people are going to access healthcare and get antibiotics: the balance between public/private/non-governmental and informal provision and between primary, secondary and tertiary care, together with the collection and disposal of unused antibiotics. In some countries, there may be extensive use in companion animals or use of human medicines for animals and vice versa. Understanding this human–animal interface is important.

In the environment, if the aim is to limit the development of AMR, the first priority would be to identify where the risks of pollution are likely to be highest, usually from the antibiotic manufacturing industry, healthcare facilities, agrofood production, or municipal/household, which are amenable to preventative and management actions ( Larsson & Flach, 2022; UNEP, 2023). Alternatively, monitoring antibiotics in sewage may offer a valuable method for estimating antibiotic use, particularly when other sources of data are scarce or unreliable ( Osena *et al.*, 2025). While sewage surveillance is cost-efficient and less prone to certain biases, it has limitations: it cannot feasibly cover all antibiotic types and lacks granular details, such as by patient group or facility, available from clinical records.

Effective environmental surveillance requires representative and harmonized data to support meaningful comparisons and evidence-based decisions. There is an urgent need for standardised methods for monitoring, sampling, measurement, as well as analysis and management of data, particularly given the focus that is now placed on this in the second Global Action Plan for AMR. This can be used to guide risk-reduction decisions and create effective incentives to follow such guidance.

For plants, it will be important to look at the production practices of the at-risk crops and to review the legality of use and alternatives to minimise the chemical inputs and their discharges into the environment ( Verhaegen *et al.*, 2023). Pesticide laws in many countries only allow products to be imported, sold or used if they have been expressly approved and in many low-resource countries, antibiotic-based pesticides are not on the approval list. Furthermore, additional regulations such as those harmonised and in the implementation stage across ECOWAS countries, require registration of pesticides to be balanced against ‘risk to humans’ and additionally the environment ( ECOWAS, 2008). Where there are such restrictions, it may still be useful to understand if use is occurring, without legitimizing such practices.

Systems to monitor antibiotic use should build upon and complement data that is being submitted to ANIMUSE (WOAH) and GLASS AMU (WHO). Nearly all countries report annually on their policies and systems to address AMR across sectors through the Tracking AMR Country Self-Assessment Survey (TrACSS) (see https://www.amrcountryprogress.org/#/map-view . While responses are subject to the perspective of those tasked with completing the survey, which may not reflect the full intended multisectoral perspective, the findings provide a starting point. Data collected might also be complemented by information on livestock and fisheries production from national sources and international sources, such as FAOSTAT and WAHIS. This can be particularly important, as denominators are required to interpret data and calculate rates of usage.

National Health Accounts can provide an indication of the relative sizes of public, private and other sectors (such as security) and of primary vs. secondary care within the overall human health system. Data on the availability of medicines and stockouts may already being collected in health facility assessment tools SARA (service readiness and assessment (SARA) and SPA Service provision assessment tools.

***Data for Action***

The priority for active monitoring should be to catalyse and inform action. This should focus and inform which data are collected, from which sources and how data are analysed ( Figure 2).

Decisions on priorities can be informed by the following questions:

• Is data already being submitted to GLASS, ANIMUSE or InFARM? What other sources of data – such as PPS—are available?
• Are these data good enough/sufficiently granular to inform and monitor action?
• If not, how could it be improved?
• What targets has the country committed to? (e.g., AWaRe index, reductions in use in food production, phasing out of growth promotion)
• Can this be monitored through current systems? (If not how will this happen, can the system also generate data for advocacy?)
• What actions are envisaged to improve use?
• Are they likely to be sustained?
• Will the data generated make a difference?
• What data are needed to monitor and refine these actions?

The initial landscape analysis would provide an indication of where actions are likely to have the greatest impact and priority actions relating to antibiotic use in the plan that should be monitored. Focusing on this, rather than increasing the complexity and number of locations, collecting data is likely to have a greater impact. In reality, there should be ongoing feedback loops, with the data generated not only being used to monitor specific actions, but also to build and refine the understanding of the antibiotic landscape and the prioritisation and focus of actions as plans are refreshed. Some countries have developed monitoring systems that were initially too comprehensive or ambitious and were not sustained after an initial period.

***Significance of Impact***

In selecting where to prioritise actions to improve and monitor use, the focus should be on how it will impact these objectives.

(Utility of Action in value chain/sector = proportion of all antibiotics used×anticipated change)/difficulty of achieving change.

In the animal sector, this focuses attention on the major value chains, but also means that “quick wins” - where significant changes can be easily achieved—are also prioritised.

In the human health sector, the focus may be determined by whether the focus is on the use of all antibiotics or specific types or categories. There are also choices to be made that involve balancing the high overall volumes of use in primary care or the informal sector with the challenges in making changes at scale in these contexts. Policies to reduce inappropriate use in primary care and the private sector by even a small increment could have significant overall impact, but implementing and monitoring at scale may be more challenging.

***Significance of Data on Antibiotics***

The most important data to collect will be those that are most important for effecting change. Although actions should be informed and monitored with data, some policy change and advocacy campaigns can occur in advance of good data, but data are crucial for stewardship programmes and other activities. The priority for establishing AMU data collection could therefore be summarised as follows:

={proportion of all antibiotics used × anticipated change} × Significance of Use data x difficulty of achieving change.

For human health, even though most antibiotics are consumed in primary care, monitoring often starts in hospitals, data collection is usually easier, resistance data are often available and the rates of use of antibiotics in the WHO Watch and Reserve are the highest ( Versporten *et al.*, 2018). Data can be used to drive stewardship programmes.

In food production, this means that complying with trade and export regulations is often the catalyst for developing monitoring systems (see Box 3).

Box 3. **Bangladesh case study.**

Bangladesh started its AMU monitoring in human and veterinary medicine, which reported to WHO GLASS (from 2022) and WOAH ANIMUSE (from 2024). Early attempts (2020–2021) to collect pharmacy-level data were unsuccessful, in part because there was no prescription record or digitisation system at 0.3 million pharmacies. Instead, information was gathered about antimicrobial medicines that were dispatched by manufacturers and importers from central warehouses. Aligning the data formats from the manufacturers and importers was difficult, and a series of workshops were needed to simplify the reporting templates.

Very high consumption of antibiotics in the Watch group in humans was identified as well as extensive usage of critically important antimicrobials (CIAs), notably 3rd/4th generation cephalosporins. High use of Watch group antibiotics in human and widespread use of CIAs (especially 3rd/4th gen cephalosporins) were observed. Its use spiked during COVID-19 pandemic. In reaction, DGDA enforced several evidence-based policy decisions: red labels on antibiotic packs ( WHO, 2024b), penalties for selling over-the-counter ( Government of the People’s Republic of Bangladesh, 2023), prohibiting veterinary use of 34 antibiotics critical in human health and including AMR in the school curricula (WHO, 2024c), all indicating examples of timely use of surveillance data for action.

***Data Collection Sources***

After ranking actions by size of impact and significance of antibiotic data, the group proposed mapping out existing opportunities for data collection, collation, analysis and sharing at different points in the One Health system. Institutions/sub-entities that oversee the various monitoring dimensions and the gaps between them should be identified. Understanding the long-term policies underpinning monitoring initiatives and potential mechanisms for sharing data between sectors is vital to ensuring long-term sustainability and financial support ( Hegewisch-Taylor *et al.*, 2024). This mapping could be based on levels and institutions, as shown in Figure 1.

Key factors to be considered in relation to the sources of data are the ease of data collection, a clearly defined population or sampling frame and the ability to standardize and benchmark data.

***Ease of Collection***

There are clear advantages to using data that are already being collected and used for other purposes, such as procurement. Inclusion of data from the private sector can be more difficult if it is seen as commercially sensitive, if sharing these data offers no obvious benefit to the private practitioner, or if the monitoring remit is considered as public sector only. The priority is usually to obtain data from the formal private sector (hospitals or farms), as reporting can be mandated through regulation or incorporated into data sharing agreements as a part of public–private partnerships. This is most efficiently achieved if it is based on automated systems that collect and collate data that already exist to avoid excess transaction costs. The Brazil case study (see Supplemental File: Annex) provides an example of how digital systems and mandatory reporting have been used. In some countries, voluntary reporting and benchmarking have also been effective. For example, the UK Red Tractor scheme provides assurance to consumers desiring high-quality food production systems with responsible use of antibiotics. Consumer pressure can be a potent incentive for private providers to comply with restrictions on use.

Academic networks often provide high-quality data and capacity to facilitate analysis. However, this is reliant on a good relationship between academia and the government.

The complexity of antibiotic use data, the need to make data extraction easy and the need to collect and analyse data from multiple sources mean that digital systems have great potential. Digitalisation of data systems enhances the potential to collect, collate and use routine data in line with countries’ data protection and use policies, but this potential will only be realised with tools to extract and analyse data in a standardised format to facilitate benchmarking. There is a risk that unless there is explicit provision to facilitate this, data on use can be extremely difficult to access and use.

***Standardised Information***

There are many different types of antibiotics, in different formulations, and data can be collected in different ways: (volume of active ingredient, numbers of doses, packs etc). Using standard metrics and approaches to collection increases potential data quality, use and scope for comparison and benchmarking ( FAO & WOAH, 2023).

***A clear sampling frame/representativeness***

To interpret and use data, there needs to be a sampling frame or reliable denominator showing the population being covered through the channel being monitored. This can be easy for a farm or hospital, or where national data are being used, but it is more challenging for retail sales, where there may be several outlets covering a given population.

***Replicability and Benchmarking***

Interpreting data on antibiotic use is difficult if there are no standards, comparators, or benchmarks. Therefore, there are clear benefits in establishing a monitoring system for a standard population that can be benchmarked and where systems can be replicated and taken to scale. Benchmarking allows the identification of trends and unusual patterns of consumption. Timely feedback to users can be useful to them in identifying where and how practice could be improved ( Yang *et al.*, 2023).

***Frequency***

Collecting and analysing data has costs and the monitoring system should consider the most efficient timing of data collection. Data generated from sources such as procurement and manufacturing should be collected on an ongoing continuous basis to develop a comprehensive picture. Where routine data sources are not available, surveys may be easier and more cost effective. Surveys can also be used to complement routine data, by providing more granular detail on how antibiotics are being used and by whom. Where data are being used to monitor compliance with regulations spot checks can be used.

Evaluating the monitoring systems can be based on tools and guidance, such as those shared by CoEvalAMR ( CoEvalAMR, 2022).

***Practical considerations for prioritisation***

Having mapped the landscape and data sources, it is also important to consider stakeholders and the relationships between them. Table 2 presents additional considerations that may shape priorities within a monitoring system as it is set up and developed.

**Table 2.** Considerations for developing antibiotic monitoring.

#	Component	Aim/objective/remark
1	Antibiotic data locations	• Understand the current places where data on antimicrobials are recorded and how these data are formulated, shared and presented. • Assess the type, quantity, and quality of data currently collected on antimicrobial distribution and use: prescription practices and compliance with treatment guidelines, antibiotic sales and supply chain data, the storage modalities of the data, paper-based or electronic. • Establish who owns data and what access requirements should be in place for the existing data to be made available for antibiotic monitoring. • Identify gaps in data coverage (e.g., rural vs. urban, private vs. public sectors) and evaluate challenges with data accuracy, timeliness, and reporting consistency. Undertake pilot studies (such as point prevalence surveys) in key locations to assess the feasiblity of proposed approaches, refine tools and validate estimates of use. • Review the potential interoperability between data systems - are standard metrics being used
2	Stakeholders	• Conduct a stakeholder mapping and analysis to delineate their relative positions, influence, interest and engagement and how they affect or could affect antibiotic monitoring. • Understanding any existing relationships and dynamics among them and their nature (formal or informal) is important for shaping and following the actions. • Recognise or acknowledge opportunities for collaboration within and across sectors under the one-health paradigm. • Identify their interests in data on AMU, what data they need and in what format to effect change.
3	Policy context including regulatory and policy framework and culture	• To elucidate key tangible and intangible influences of implementation of antibiotic monitoring. Such influences inform available opportunities, enablers, barriers and limits to processes such data collection, sharing, analysis, reporting and action on these data. • The *policy context* includes the regulatory and policy framework including the extent of their implementation, their strengths and weaknesses, existing technical expertise and know-how, the relationships and dynamics of among the key stakeholders, social, cultural and behavioural factors such as levels of trust and willingness to share antimicrobial data and potential incentives or barriers to behaviour change.
4	Readiness for and sustainability of implementation of antibiotic monitoring	• To delineate the available resources, commitment, and motivation that can be leveraged towards antibiotic monitoring • The resources include infrastructure such as information communication technology, office space and other data management infrastructure, material and human resources. • The willingness for collaboration between government and academia and with the private sector. • This component usually includes assessment of existing systems or sub-systems for antibiotic monitoring with the aim of identifying gaps, inefficiencies, areas of overlap, potential for some to be carried forward, strengthened and scaled up, possibility of integration with other components such as medicines management, AMR data, environmental sampling or scaling up to other units and facilities. • Assess embedded strategies for long-term sustainability to ensure the continued function and expansion of monitoring where needed.

***Getting started***

In the initial phases of setting up systems to monitor antibiotics, it may be best to focus on areas known to be important to the development and spread of resistance and where data capture is straightforward.

***Coordination: building a system***

There was consensus that merely collecting data is not enough and that analysis should create information that drives action and generates information on the effectiveness of that action. Effective communication and coordination among those generating, analysing and using data is vital. This should mean that the data generated from a monitoring system reflects the priorities for action, provides useful and timely information and that the monitoring system evolves with the overall strategy and plans for optimising use. Monitoring in an individual facility - a hospital, health centre, or farm–can generate useful information and inform action at the local level; however, when the scope and ambition are wider, it is important to have a system and coordination. Often, people using data are not the same as those supplying it. A coordinated system facilitates benchmarking and representative sampling. This can facilitate links between different users of data in private, public and faith-based sectors.

For data to be used effectively, it should ideally reflect and respond to the interests and priorities and be in a format that is useful to each organisation. Relationships and power dynamics between stakeholders can be complex and it may be helpful to refer back to the stakeholder analysis to decide how and with whom information should be shared. A structured process that considers antibiotic monitoring priorities in light of AMR data could be useful, and could follow processes that have been used to prioritise pathogens for AMR surveillance ( Patel *et al*. 2022).

Given the complexities of data collection and analysis, there will likely be errors, gaps and overlaps in the initial rounds. Good communication and prompt non-judgemental feedback can help identify and rectify these issues and strengthen the system. Although collaboration across sectors is important, getting effective and inclusive systems and structures for collaboration within sectors is vital. In human health, encouraging collaboration and data sharing and use between public, private, academic and faith-based institutions is challenging, but can result in a more robust system.

Given that there are many potential users of data on antibiotic use, a consolidated national report on antibiotic use will be useful, whether as a standalone document on medicines, in conjunction with data on antibiotic resistance (as in Thailand) or as a chapter of the report on NAP implementation.

Examples from different sectors and settings illustrate how steps towards national monitoring systems have been taken with different challenges and solutions in mind. Box 4 summarises brief insights from the setting up of antibiotic monitoring in Malawi and Chile.

Box 4. **Cases illustrating the development of antimicrobial monitoring systems in different contexts.**

**Malawi example.**

In response to a call by WOAH for Members to submit data on antimicrobial use, Malawi began collecting antimicrobial consumption data from agrovet shops. This initiative is coordinated by the Department of Animal Health and Livestock Development, and the data is now submitted to the ANIMUSE platform. The purpose is to better understand national antimicrobial usage patterns in animals and support evidence-based decision-making at the country level.

In response to recognition that the resistance pattern in humans was shown to vary in different regions for example *e.coli* and salmonella had higher resistance in the southern part, compared to the northern part, the National AMR Core Group wanted to explore whether this was linked to differences in use of antibiotics in animals. The team recruited final year veterinary students who produced interesting data on differences between local village chicken AMR/AMU and that seen in broilers. This case showed low use of antimicrobials and low antimicrobial resistance to commonly used antibiotics in local village chickens compared to the broiler chickens. This could be attributed to the fact that broilers are usually raised under intensive management systems where antibiotics are heavily used. The case also supported a pathway for capacity strengthening.

**Chile example.**

In Chile, the antimicrobial medicines monitoring system for aquaculture is more developed than for terrestrial animals. Since 2018 an electronic prescription system has been implemented, as a joint initiative between the national fisheries service and the laboratory FARMAVET from the University of Chile. The system means that each time an antimicrobial is administered it must have a prescribed treatment with information, declaration of the veterinarian, and a record of the cage, species and diagnosis. This is included in a real-time electronic system. For terrestrial animals, also an electronic prescribing system from the national Livestock and agricultural service is in place since 2023. To date it is mandatory for purchase of antimicrobials and for companion animals but voluntary for prescription and declaration of production animals. The next step will be to have mandatory electronic prescription for all animal productive species, and pets, in order to have the data of purchase and use. For the implementation of both systems, the public private partnership, and the collaborative work between government, private sector and academia, has been fundamental.

## Conclusions

Antibiotics are essential to human and animal healthcare and integral to modern systems of animal and plant production. Balancing affordable access with the need to preserve effectiveness – by reducing infection burden, curbing misuse and promoting safe disposal – remains a significant challenge. Stockouts increase costs and compromise present health outcomes, while excess and misuse fuel drug resistance and harm long-term outcomes. Effective medicines management requires data on antibiotic volumes, types and locations to be analysed and made rapidly available to decision-makers at local, regional and national levels; however, gathering this data demands a complex and costly system. This paper describes available tools for collecting, analysing and sharing antibiotic data across sectors, and sets out principles to prioritise these activities according to their potential impact.

The overarching principle proposed is ***action-oriented prioritisation***: investing in the components of a monitoring system most likely to drive action with a net positive impact, as recently highlighted for environmental AMR-surveillance ( Larsson *et al*., 2026). Prioritisation should be guided by an assessment of the scale and nature of problematic antibiotic manufacturing, distribution and use, informed by agreed targets in a National Action Plan on AMR and other national plans, including those for Universal Health Coverage. Within this framework, we propose three foundational approaches: a **risk-based approach** that focuses monitoring efforts on the most critical threats to human and animal health and high-risk antibiotic residues in the environment; a **sustainable approach** that builds local expertise, infrastructure and technology integrated with broader country systems; and a **transparent system** that makes evidence available at the local level, with feedback on actions taken in response.

Effective cross-sector and cross-level coordination is essential. Data from an individual facility or farm must serve that institution while also being available – through a managed system – for use by other sectors and at other levels. For data to be meaningful, collection and analysis should follow standard indicators (e.g., ATC, DDDs, mg/kg and AWaRe), with a clearly defined sampling frame covering relevant population groups and geographies. Collection frequency should reflect whether more or less regular analysis would support different actions. A sound understanding of the existing activity landscape, key stakeholders, data interests and policy constraints is essential before additional investment is made. Emerging opportunities should also be planned for: digitisation can greatly facilitate data collection when standardised approaches are embedded from the outset, and regulated e-pharmacy and online medicine sales could simplify data collection through mandatory reporting.

In any country, developing an antibiotic monitoring system incrementally is likely to require both a top-down approach – linking to global systems such as ANIMUSE and GLASS – and a bottom-up approach that identifies priority areas for action to drive local and national change. In both cases, data should be collected, classified and analysed in a standard format (e.g., ATC, ATCvet, active ingredients/molecules and AWaRe) to enable benchmarking and cross-country learning. Appropriate denominators aid comparison: GLASS uses DDDs, while ANIMUSE uses mg/kg animal biomass. Coordination between levels is critical, as data collected at one level may require action at another, and communication across sectors is equally important for sharing evidence and enabling action at all levels.

Sustaining systems and securing long-term funding is challenging, but systems that generate actionable insights that are useful for farmers and veterinarians, clinicians, policymakers and procurement specialists, as well as contributing to global policy, are more likely to receive ongoing investment.

Comprehensively monitoring antibiotic use across all systems, sectors, molecules and routes of administration is challenging with limited resources. Targeting monitoring efforts towards higher-risk, higher-impact areas – while ensuring inclusion of all potential data sources, integration with national agendas and transparency in evidence-sharing – will support sustainability and provide a strategic path to the most essential components of an antibiotic monitoring system that can inform specific actions on antibiotic access, AMR and antibiotic residues in food and the environment.

### Data availability statement

There were no data generated in this project. Proceedings from the working group can be found in the supplementary file.

## Supplementary file

Valsson *et al.* (2025) Antibiotic surveillance: an action-oriented integrated approach. SEDRIC Working Group Inventory, Proceedings and Annex. DOI: https://doi.org/10.17037/PUBS.04677845. Data are available under the terms of the Creative Commons Attribution 4.0 International license (CC-BY 4.0).
