# Supplementary material for: Strengthening systems to monitor antibiotics. An action oriented approach
Source: Wellcome Open Res. 2026 Sep 24;11:55. Originally published 2026 Jan 23. [Version 2] doi: 10.12688/wellcomeopenres.24568.2 (PMC13126008; doi:10.12688/wellcomeopenres.24568.2)
Supplement: Peer review history

# Reviewer report: Reviewer response for version 1

**DOI:** 10.21956/wellcomeopenres.27077.r151876

**Published:** 2026-04-28

**Authors:** Teresa Wozniak

**Affiliations:** 1 The Australian e-Health Research Centre, CSIRO, Qld, Australia

This manuscript addresses an important and timely topic, and reflects effort in bringing together perspectives across sectors. While the overall content is valuable, particularly in aiming to support action on AMR , there are several areas where the clarity, consistency, and methodological transparency of the paper needs to be strengthened. In particular, clearer definitions/ use of language, a more detailed description of the methods and most importantly synthesis of the evidence are needed to improve the credibility and usability of these findings. The comments below are for each section of the manuscript

 Abstract:

- method : state regions represented not just ‘world’; state number of workshops and meetings
- explain the action-orientation prioritisation methodology
- word ‘effort’ duplicated ..to inform efforts to mitigate antimicrobial resistance (AMR) mitigation efforts and beyond

Introduction

- is there a reference for plant production accelerating the emergence and spread of resistant microorganisms ?
- Reference ‘ Papst L, Luzzati R, Carević B, *et al.*: Antimicrobial use in hospitalised patients with COVID-19: an international multicentre point-prevalence study. *Antibiotics (Basel).* 2022; **11**(2): 176.” should not be used. The study does not have a methodology section and it’s a known predatory journal. suggest other references to make this point
- Please provide a reference to the specific naming of ‘terrestrial and aquatic animals’ , the other sections only refer to animals.
- What is ‘antimicrobial medicine surveillance’? Is this antimicrobial use or appropriateness or both?  suggest to use commonly known language. This is also referred to in Findings where authors state ‘ value of antibiotic surveillance’ and use AMU acronym.  Suggest to be consistent with language use

Methods

- This section should state the methodology in more detail and include information on the regions where the experts came from, their expertise and experience. Stating they are SEDRIC reps and consultants doesn’t provide sufficient detail and validation of their expertise. Good to include organisations as has been done
- How many people per One Health domain area?
- Literature review need to be expanded and include dates, search approach/ strategy, synthesis approach
- The last paragraph starting ‘ This paper is aimed…’ more likely should be in the introduction section rather than methodology.

Findings

 This section would benefit from more of a synthesis of the workshops, meetings and literature review that were undertaken as part of the study. In some sections  its quite vague and does not refer to who / which sector contributed to  which parts of the finding. It needs to be backed up by some level of traceability in decision making and or evidence of why this is needed. At the moment, it reads like a literature review but as the methodology section was not clear about how this was conducted, I am still unsure if its comprehensive and complete. This section needs major review and would benefit from being more succinct.

 Below are specific comments related to this section:

- Value of antibiotic surveillance – what does this include? Box 1 refers to AMU data , is that what is meant what about appropriateness? it could even be confused with AMR surveillance.
- AMU included but not spelled out

- how was consensus reached? this need to be described in methods and then explained in findings.
- Figure 1 is really great but would add into the key of tools 1. GLASS etc. the countries that are covered or if global such as GLASS then state that.  in the same figure how was the complexity determined in the legend? is this something that the authors assessed as part of the study or was complexity of data collection validated prior to the study?
- Please provide a reference for this ‘Although antibiotic use in plants is estimated to be lower than that in humans and animals’
- please spell out all acronyms ie FAOSTAT
- Reword these two sentence, as it unclear what is meant ‘This would allow sentinel surveillance to understand the nature of use and allow research for alternatives promoting initiatives such as integrated pest management and other practices to reduce the use and discharge into the environment. At the national level, it may be possible to track the quantities used as pesticides.’
- statements such as this ’Formalized environmental surveillance of antibiotics remains limited. ‘ will need to be backed up by more detail, its this global or specific to a particular country, region, income setting.
- Table 1 – solution under animal sector is strengthen AMR surveillance , is that meant to read AU ? I don’t see much of a discussion on AMR surveillance in animals. Also table 1 is mentioned much later than it fist appears and the table is not described in much detail.
- data are plural so should read as “what data are being collected”
- what is the definition of ‘fully integrated surveillance system”? this can mean so many different things and needs a definition here for this study. It should be more specific than stating ‘ connecting all systems and incorporating all sources of data and data collection tools”
- the steps in prioritisation which are the main finding of this paper require more details as I have described above, how have authors come to these steps of prioritisation? how was consensus reached, by how many people . this paragraph is again very generic and somewhat limited of any form of evidence of data synthesis.
- synthesis of all sections in findings need to be detailed and how authors came to make these conclusions.

Conclusions

- suggest to be more succinct here.
- I am unsure why some words were highlighted
- Comment such as ‘….our analysis shows that effective coordination is critical  ‘ I don’t think the current manuscript actually shows any analysis and I would recommend strongly that this is included.
- AMU is now mentioned in the final conclusion, clarity on what is proposed to be surveyed is needed.This manuscript addresses an important and timely topic, and reflects effort in bringing together perspectives across sectors. While the overall content is valuable, particularly in aiming to support action on AMR , there are several areas where the clarity, consistency, and methodological transparency of the paper needs to be strengthened. In particular, clearer definitions/ use of language, a more detailed description of the methods and most importantly synthesis of the evidence are needed to improve the credibility and usability of these findings. The comments below are for each section of the manuscript
  Abstract:
- method : state regions represented not just ‘world’; state number of workshops and meetings
- explain the action-orientation prioritisation methodology
- word ‘effort’ duplicated ..to inform efforts to mitigate antimicrobial resistance (AMR) mitigation efforts and beyond
- Introduction
- is there a reference for plant production accelerating the emergence and spread of resistant microorganisms ?
- Reference ‘ Papst L, Luzzati R, Carević B, *et al.*: Antimicrobial use in hospitalised patients with COVID-19: an international multicentre point-prevalence study. *Antibiotics (Basel).* 2022; **11**(2): 176.” should not be used. The study does not have a methodology section and it’s a known predatory journal. suggest other references to make this point
- Please provide a reference to the specific naming of ‘terrestrial and aquatic animals’ , the other sections only refer to animals.
- What is ‘antimicrobial medicine surveillance’? Is this antimicrobial use or appropriateness or both?  suggest to use commonly known language. This is also referred to in Findings where authors state ‘ value of antibiotic surveillance’ and use AMU acronym.  Suggest to be consistent with language use
- Methods
- This section should state the methodology in more detail and include information on the regions where the experts came from, their expertise and experience. Stating they are SEDRIC reps and consultants doesn’t provide sufficient detail and validation of their expertise. Good to include organisations as has been done
- How many people per One Health domain area?
- Literature review need to be expanded and include dates, search approach/ strategy, synthesis approach
- The last paragraph starting ‘ This paper is aimed…’ more likely should be in the introduction section rather than methodology.
- Findings
  This section would benefit from more of a synthesis of the workshops, meetings and literature review that were undertaken as part of the study. In some sections  its quite vague and does not refer to who / which sector contributed to  which parts of the finding. It needs to be backed up by some level of traceability in decision making and or evidence of why this is needed. At the moment, it reads like a literature review but as the methodology section was not clear about how this was conducted, I am still unsure if its comprehensive and complete. This section needs major review and would benefit from being more succinct.
  Below are specific comments related to this section:
- Value of antibiotic surveillance – what does this include? Box 1 refers to AMU data , is that what is meant what about appropriateness? it could even be confused with AMR surveillance.
- AMU included but not spelled out
- how was consensus reached? this need to be described in methods and then explained in findings.
- Figure 1 is really great but would add into the key of tools 1. GLASS etc. the countries that are covered or if global such as GLASS then state that.  in the same figure how was the complexity determined in the legend? is this something that the authors assessed as part of the study or was complexity of data collection validated prior to the study?
- Please provide a reference for this ‘Although antibiotic use in plants is estimated to be lower than that in humans and animals’
- please spell out all acronyms ie FAOSTAT
- Reword these two sentence, as it unclear what is meant ‘This would allow sentinel surveillance to understand the nature of use and allow research for alternatives promoting initiatives such as integrated pest management and other practices to reduce the use and discharge into the environment. At the national level, it may be possible to track the quantities used as pesticides.’
- statements such as this ’Formalized environmental surveillance of antibiotics remains limited. ‘ will need to be backed up by more detail, its this global or specific to a particular country, region, income setting.
- Table 1 – solution under animal sector is strengthen AMR surveillance , is that meant to read AU ? I don’t see much of a discussion on AMR surveillance in animals. Also table 1 is mentioned much later than it fist appears and the table is not described in much detail.
- data are plural so should read as “what data are being collected”
- what is the definition of ‘fully integrated surveillance system”? this can mean so many different things and needs a definition here for this study. It should be more specific than stating ‘ connecting all systems and incorporating all sources of data and data collection tools”
- the steps in prioritisation which are the main finding of this paper require more details as I have described above, how have authors come to these steps of prioritisation? how was consensus reached, by how many people . this paragraph is again very generic and somewhat limited of any form of evidence of data synthesis.
- synthesis of all sections in findings need to be detailed and how authors came to make these conclusions.
- Conclusions
- suggest to be more succinct here.
- I am unsure why some words were highlighted
- Comment such as ‘….our analysis shows that effective coordination is critical  ‘ I don’t think the current manuscript actually shows any analysis and I would recommend strongly that this is included.
- AMU is now mentioned in the final conclusion, clarity on what is proposed to be surveyed is needed.

Is the work clearly and accurately presented and does it cite the current literature?

Partly

If applicable, is the statistical analysis and its interpretation appropriate?

Not applicable

Are all the source data underlying the results available to ensure full reproducibility?

No

Is the study design appropriate and is the work technically sound?

Partly

Are the conclusions drawn adequately supported by the results?

Partly

Are sufficient details of methods and analysis provided to allow replication by others?

No

Reviewer Expertise:

Epidemiology, AMR surveillance and response, burden of disease studies, molecular biology

I confirm that I have read this submission and believe that I have an appropriate level of expertise to confirm that it is of an acceptable scientific standard, however I have significant reservations, as outlined above.

# Response

**Published:** 2026-09-13

**Authors:** Clare Chandler

**Affiliations:** London School of Hygiene & Tropical Medicine, UK

**Response to reviewer comments** Authors’ response: We thank the reviewer for the review and helpful comments. We have revised the manuscript in line with each of these suggestions in turn and believe this has strengthened the paper as a whole.  

 Reviewer comment: This manuscript addresses an important and timely topic, and reflects effort in bringing together perspectives across sectors. While the overall content is valuable, particularly in aiming to support action on AMR , there are several areas where the clarity, consistency, and methodological transparency of the paper needs to be strengthened. In particular, clearer definitions/ use of language, a more detailed description of the methods and most importantly synthesis of the evidence are needed to improve the credibility and usability of these findings. The comments below are for each section of the manuscript

 Authors’ response: We appreciate the positive comments of the reviewer regarding the timeliness, relevance and clarity of the paper.  We have reviewed the comments point by point and revised the manuscript in line with each and as outlined below.   Reviewer comments: Abstract:

- method : state regions represented not just ‘world’; state number of workshops and meetings. 
- explain the action-orientation prioritisation methodology.
- word ‘effort’ duplicated ..to inform efforts to mitigate antimicrobial resistance (AMR) mitigation efforts and beyond

Authors’ response: Thank you for noting the grammatical issues, which we have updated, along with refining representation. As the abstract word count is constrained, we are unfortunately unable to go into details of the methodologies, but we have expanded on this in the full text.   Reviewer comments: Introduction

- is there a reference for plant production accelerating the emergence and spread of resistant microorganisms ?
- Reference ‘ Papst L, Luzzati R, Carević B, *et al.*: Antimicrobial use in hospitalised patients with COVID-19: an international multicentre point-prevalence study. *Antibiotics (Basel).* 2022; **11**(2): 176.” should not be used. The study does not have a methodology section and it’s a known predatory journal. suggest other references to make this point.
- Please provide a reference to the specific naming of ‘terrestrial and aquatic animals’ , the other sections only refer to animals.
- What is ‘antimicrobial medicine surveillance’? Is this antimicrobial use or appropriateness or both?  suggest to use commonly known language. This is also referred to in Findings where authors state ‘ value of antibiotic surveillance’ and use AMU acronym.  Suggest to be consistent with language use.

Authors’ response:

- Thank you for noting this and we reworded the sentence to reflect the lack of data to conclude on the relationship between antimicrobial use in plants and the development of AMR in humans and animals.
- We have deleted the reference on antibiotic use in hospitalised Covid patients as suggested, particularly as with the passing of time this becomes less pertinent.
- We have clarified in the introduction that ‘animals’ in this manuscript refers both to terrestrial and aquatic, unless the specific classification applies.
- We noted the confusion from all reviewers arising from the term antibiotic surveillance, and have amended title and text refer to antibiotic monitoring instead.  We have clarified this and also attempted to ensure more consistency in terminology and definitions throughout.

  Reviewer comments: Methods

- This section should state the methodology in more detail and include information on the regions where the experts came from, their expertise and experience. Stating they are SEDRIC reps and consultants doesn’t provide sufficient detail and validation of their expertise. Good to include organisations as has been done. 
- How many people per One Health domain area?
* *
- Literature review need to be expanded and include dates, search approach/ strategy, synthesis approach. 
- The last paragraph starting ‘ This paper is aimed…’ more likely should be in the introduction section rather than methodology. 

Authors’ response: We have expanded and clarified the methodology section to detail the expertise of individuals concerned, and the steps and dates in the process, and shifted the final paragraph to the introduction section as suggested.  

 Reviewer comment: Findings

 This section would benefit from more of a synthesis of the workshops, meetings and literature review that were undertaken as part of the study. In some sections  its quite vague and does not refer to who / which sector contributed to  which parts of the finding. It needs to be backed up by some level of traceability in decision making and or evidence of why this is needed. At the moment, it reads like a literature review but as the methodology section was not clear about how this was conducted, I am still unsure if its comprehensive and complete. This section needs major review and would benefit from being more succinct.**

 Authors’ response: We hope that expansion of the methods section has gone some way to addressing this concern and in addition have revised the findings sections to be more succinct and to address the specific comments from the reviewer below.   Reviewer comments: Below are specific comments related to this section:

- Value of antibiotic surveillance – what does this include? Box 1 refers to AMU data, is that what is meant what about appropriateness? it could even be confused with AMR surveillance. 
- AMU included but not spelled out.
- how was consensus reached? this need to be described in methods and then explained in findings. 
- Figure 1 is really great but would add into the key of tools 1. GLASS etc. the countries that are covered or if global such as GLASS then state that.  in the same figure how was the complexity determined in the legend? is this something that the authors assessed as part of the study or was complexity of data collection validated prior to the study?  

Authors’ response:

- We have spelled out AMU and revised the text to clarify the distinction between AMU and AMR surveillance, which we agree can be confusing.
- The process for consensus is now explained in the methods and results. The key point of consensus from the group was prioritisation of data collection and analysis that would have a chance to inform action. This point emerged and solidified through the in-person workshop and was followed up for agreement with those participants and subsequent contributors in the reference group.
- We appreciate the positive comments about Figure 1.  We note the reviewer’s request to add which countries are covered by which sorts of tools. However, these data are extremely difficult to source accurately and keep up to date, particularly as some countries may undertake monitoring in just one locality or for a specific time period. Complexity of tools was based on the discussions with individual experts and the conclusions of meetings. The tools are referred to in the boxes by numbers.

  Reviewer comments:

- Please provide a reference for this ‘Although antibiotic use in plants is estimated to be lower than that in humans and animals’

- please spell out all acronyms ie FAOSTAT.
- Reword these two sentence, as it unclear what is meant ‘This would allow sentinel surveillance to understand the nature of use and allow research for alternatives promoting initiatives such as integrated pest management and other practices to reduce the use and discharge into the environment. At the national level, it may be possible to track the quantities used as pesticides.’
- statements such as this ’Formalized environmental surveillance of antibiotics remains limited. ‘ will need to be backed up by more detail, its this global or specific to a particular country, region, income setting. 

Authors’ response:

- We have added references and a reworded the sentence on the volume of antibiotics used in plants.
- FAOSTAT is not an acronym, but the name of the FAO statistical database.  Other acronyms have been spelt out in full, when first used.
- We have replaced the sentences on surveillance for pest management as follows, “This questionnaire is intended to gather information on the use of antibiotics in plants from both quantitative and qualitative perspectives. Data collected include the amounts of antibiotics used, types of antibiotics used and authorized, crops treated, targeted plant diseases, and the application practices. Collectively, these data will provide a more comprehensive understanding of how antibiotics are used in plants and the factors influencing their use.”
- We have revised the manuscript to ensure clarity of specific instances of data limitations where needed.

  Reviewer comments:

- Table 1 – solution under animal sector is strengthen AMR surveillance , is that meant to read AU ? I don’t see much of a discussion on AMR surveillance in animals. Also table 1 is mentioned much later than it fist appears and the table is not described in much detail. 
- data are plural so should read as “what data are being collected”
- what is the definition of ‘fully integrated surveillance system”? this can mean so many different things and needs a definition here for this study. It should be more specific than stating ‘ connecting all systems and incorporating all sources of data and data collection tools”.

Authors’ response:

- Table 1 did have a typo which has been corrected.  This paper is about the surveillance or monitoring of antibiotic use, but to clarify this we have changed to monitoring throughout, and monitoring resistance in animals is not within scope.
- We have reviewed the manuscript to ensure consistent grammar relating to data.
- We have clarified ‘fully integrated surveillance system’ to a comprehensive surveillance system being informed by all available sources.

  Reviewer comments:

- the steps in prioritisation which are the main finding of this paper require more details as I have described above, how have authors come to these steps of prioritisation? how was consensus reached, by how many people . this paragraph is again very generic and somewhat limited of any form of evidence of data synthesis.
- synthesis of all sections in findings need to be detailed and how authors came to make these conclusions.

Conclusions

- suggest to be more succinct here.
* *
- I am unsure why some words were highlighted
- Comment such as ‘….our analysis shows that effective coordination is critical  ‘ I don’t think the current manuscript actually shows any analysis and I would recommend strongly that this is included.
- AMU is now mentioned in the final conclusion, clarity on what is proposed to be surveyed is needed. 

Authors’ response:

- The steps to prioritisation reflect a logical approach, which evolved through workshopping with the technical working group and was refined iteratively as the paper was finalised.  The same approach was used to reach other conclusions. This is now clarified in the text.
- The consensus issue is now addressed earlier in the paper.
- The conclusions have been revised to be more succinct.
- The words that are indicated in bold are called out for readers as key issues arising for action from the article.
- The phrasing around the analysis of effective coordination has been edited.
- The wording around AMU has been revisited throughout, to improve clarity on the focus as antibiotic monitoring.

  Once again, we are very grateful to the reviewer for taking the time and effort to review our manuscript and provide constructive feedback. We hope they will agree that the manuscript is strengthened as a result.

# Reviewer report: Reviewer response for version 1

**DOI:** 10.21956/wellcomeopenres.27077.r151878

**Published:** 2026-04-09

**Authors:** Olabisi Promise Lawal

**Affiliations:** 1 University of Benin, Benin City, Nigeria

The manuscript presents a One Health–oriented, action-focused framework for antibiotic surveillance in resource-constrained settings, combining literature review, expert consultation, and workshop inputs to propose a prioritization approach for feasible and impactful data collection. The topic is timely and relevant, and the paper offers practical value for policymakers, particularly in low- and middle-income countries.

 The work is clearly written and engages with relevant literature, but only partly achieves depth in critical analysis, as most existing tools are described rather than rigorously compared. The study design is appropriate for a conceptual and policy-oriented paper; however, it lacks sufficient methodological transparency to be considered fully technically sound.

 In particular, the literature review process, expert selection, and analytical approach are not described in enough detail, making replication difficult. For this reason, the methods and analysis are only partly adequate for reproducibility.

 Statistical analysis is not applicable, as the study is primarily qualitative. Source data are partly available, as the work relies on secondary literature and expert input, but no structured dataset or supplementary material is provided. The conclusions are generally supported by the arguments presented, though they would benefit from stronger empirical grounding.

 To make the article scientifically sound, several key issues must be addressed. The authors need to provide a clear and reproducible methodology, including search strategy, inclusion criteria, and how expert input was gathered and synthesized. The paper would also benefit from a more critical comparison of existing surveillance tools, highlighting their strengths, limitations, and applicability across contexts. In addition, the proposed framework is not sufficiently validated; the authors should either strengthen this through more detailed case applications or clearly position it as a conceptual model with future validation needs.

 Expanding the discussion to include limitations, trade-offs, and positioning within existing frameworks would further strengthen the manuscript.

 Overall, the paper has strong potential, but requires substantial revision to improve methodological rigor, analytical depth, and clarity of contribution.

Is the work clearly and accurately presented and does it cite the current literature?

Yes

If applicable, is the statistical analysis and its interpretation appropriate?

Yes

Are all the source data underlying the results available to ensure full reproducibility?

Yes

Is the study design appropriate and is the work technically sound?

Yes

Are the conclusions drawn adequately supported by the results?

Yes

Are sufficient details of methods and analysis provided to allow replication by others?

Yes

Reviewer Expertise:

Antimicrobioal resistance, Aging,Gerontology,Public health

I confirm that I have read this submission and believe that I have an appropriate level of expertise to confirm that it is of an acceptable scientific standard.

# Response

**Published:** 2026-09-13

**Authors:** Clare Chandler

**Affiliations:** London School of Hygiene & Tropical Medicine, UK

**Response to reviewer comments**

 Authors’ response: We thank the reviewer for the review and helpful comments. We have revised the manuscript in line with each of these suggestions in turn and believe this has strengthened the paper as a whole.  

 Reviewer comment: The manuscript presents a One Health–oriented, action-focused framework for antibiotic surveillance in resource-constrained settings, combining literature review, expert consultation, and workshop inputs to propose a prioritization approach for feasible and impactful data collection. The topic is timely and relevant, and the paper offers practical value for policymakers, particularly in low- and middle-income countries.

 Authors’ response: We appreciate the positive comments of the reviewer, regarding the timeliness, relevance and clarity of the paper.  

 Reviewer comment: The work is clearly written and engages with relevant literature, but only partly achieves depth in critical analysis, as most existing tools are described rather than rigorously compared. The study design is appropriate for a conceptual and policy-oriented paper; however, it lacks sufficient methodological transparency to be considered fully technically sound.

 In particular, the literature review process, expert selection, and analytical approach are not described in enough detail, making replication difficult. For this reason, the methods and analysis are only partly adequate for reproducibility.

 Authors’ response: We have noted the comments about methodology and have amended the text to include more detail of the consultative process, and snowball methodology to identify sources, (which are largely in the grey literature and not amenable to conventional literature reviews). The framework that is proposed is a logical proposal and consensus view of a group of experts from a wide range of geographical contexts and sectoral experiences.  It has not yet been validated through widespread adoption.

 Reviewer comment: Statistical analysis is not applicable, as the study is primarily qualitative. Source data are partly available, as the work relies on secondary literature and expert input, but no structured dataset or supplementary material is provided. The conclusions are generally supported by the arguments presented, though they would benefit from stronger empirical grounding.

 Authors’ response: There are limited studies available that evaluate the use of different guidelines and tools for antibiotic monitoring, and therefore expert input from a range of geographical contexts was considered most appropriate to move this field forwards.

 Reviewer comment: To make the article scientifically sound, several key issues must be addressed. The authors need to provide a clear and reproducible methodology, including search strategy, inclusion criteria, and how expert input was gathered and synthesized. The paper would also benefit from a more critical comparison of existing surveillance tools, highlighting their strengths, limitations, and applicability across contexts. In addition, the proposed framework is not sufficiently validated; the authors should either strengthen this through more detailed case applications or clearly position it as a conceptual model with future validation needs.

 Authors’ response: There is significant discussion in the paper about other tools and frameworks – and further expanding this would run counter to the advice of other reviewers that the paper is already long. 

 Reviewer comment: Expanding the discussion to include limitations, trade-offs, and positioning within existing frameworks would further strengthen the manuscript.

 Authors’ response: Given the importance of context, it is difficult to generalise on strengths and weakness at a global level. The discussion positions the effort to monitor antimicrobials within existing frameworks.

   Reviewer comment: Overall, the paper has strong potential, but requires substantial revision to improve methodological rigor, analytical depth, and clarity of contribution. Authors’ response: We have revised the paper to provide more information on the methods and analysis and to enhance clarity. Once again, we are very grateful to the reviewer for taking the time and effort to review our manuscript and provide constructive feedback. We hope they will agree that the manuscript is strengthened as a result.

# Reviewer report: Reviewer response for version 1

**DOI:** 10.21956/wellcomeopenres.27077.r145869

**Published:** 2026-03-12

**Authors:** Lucie Collineau

**Affiliations:** 1 Epidemiology and Surveillance Support Unit, University of Lyon - French Agency for Food, Environmental and Occupational Health and Safety (ANSES), Lyon, France

Major comments

 This manuscript aims to provide guidance on antibiotic surveillance, specifically to support prioritization of surveillance activities within countries (or settings) with limited resources. The information provided is generally comprehensive and accurate, and the authors make an interesting point that antibiotic surveillance activities should be prioritized to feed into actions.

 Most of the content is not new, so I would rather classify this manuscript as a review, rather than a research article.

 The text is very long, and could be made more concise by referring to previous similar reviews, e.g.

 Refer to reference 1, 2 & 3

 The authors should define the scope of “antibiotic surveillance” retained in this study; most of the content relates to surveillance of antibiotic use (and antibiotic residues, to some extent). However, surveillance of AMR in itself (and how to prioritize it) is not really covered. Maybe the title of the paper should be clarified accordingly.

 Several important initiatives are missing, such as the FAO Progressive Management Pathway for Antimicrobial Resistance (PMP-AMR), and the Quadripartite Technical Group on Integrated Surveillance on antimicrobial use and resistance (QTG-AIS).

 Description or recommendations related to environmental surveillance are rather sparse. It seems that the environmental sector was not really represented in the technical working group that worked on this study. Maybe a limitation to be mentioned in the text?

 Similarly, information is rather limited about AMU surveillance in plants (page 14); maybe similar tools that those used in animal farming could be used?

 I suggest the supplementary file that lists ongoing surveillance initiatives to focus only on global programmes. In the current version, there are a few examples from national surveillance systems as well (e.g. NARMS, German systems, etc.) , but these are nor comprehensive nor illustrative of LMIC countries, so it is a bit unclear how they were selected and why they are of interest here. They make the scope of the paper a bit fuzzy. However, examples from LMIC in the core text (as text boxes) are welcome.

 Please harmonize the terminology throughout: sector vs domain

 Please limit the number of acronyms to the strict minimum; Page 15: what does SF means?

 Minor comments

 About AMR surveillance in the environment (page 11): maybe mention the new EU directive that will make AMR surveillance in wastewater mandatory in large cities (>100 000 inhab).

 About engagement of local actors: not only regulation can facilitate data submission; voluntary win-win systems can be very effective and sustainable too

 Page 14: with AI and big data becoming a growing field of research, probably the data collection tools can adapt to the data sources, rather than the other way around.

 Page 15: the primary goal of AM residues monitoring is to protect the consumer from chemical risks related to residues in food (not to monitor AMU or illegal use); please edit

 Page 16: any other attempt than JIACRA to compare AMU between sectors?

 Table 1: the focus is on AMU only, please edit the title accordingly. An important gap for environmental surveillance is to select appropriate indicators; this is work in progress. Consider expanding animal health tools to plant farming? Who are the actors in charge of ‘prescribing’ AMU in plants?

 Page 18: calculate rates of usage

 Figure 3: consider adding field experts (e.g. water agencies, etc.) on environmental surveillance (not only the ministry). It seems primary care professionals are missing in this table. Not sure AMU on prescription only is feasible in certain LMIC where veterinary services are scarce. Add NGOs and international organizations as well?

 Page 20: add InFARM? Together with GLASS and ANIMUSE

 Table 2: consider adding pilot studies or point prevalence surveys, which are often very good starting points before building sustainable surveillance systems. They can also help to prioritize surveillance efforts by identifying high-risk populations or sectors. The way the table is presented is maybe over-ambitious (comprehensive mapping of all data sources, actors, etc.). Better start small and then expand.

Is the work clearly and accurately presented and does it cite the current literature?

Partly

If applicable, is the statistical analysis and its interpretation appropriate?

Not applicable

Are all the source data underlying the results available to ensure full reproducibility?

Yes

Is the study design appropriate and is the work technically sound?

Yes

Are the conclusions drawn adequately supported by the results?

Yes

Are sufficient details of methods and analysis provided to allow replication by others?

Yes

Reviewer Expertise:

veterinary epidemiology

I confirm that I have read this submission and believe that I have an appropriate level of expertise to confirm that it is of an acceptable scientific standard, however I have significant reservations, as outlined above.

## References

[1] : EPI-Net One Health reporting guideline for antimicrobial consumption and resistance surveillance data: a Delphi approach. *The Lancet Regional Health - Europe* .2023;26: 10.1016/j.lanepe.2022.100563 10.1016/j.lanepe.2022.100563 PMC998963236895445

[2] : Monitoring Antimicrobial Resistance and Drug Usage in the Human and Livestock Sector and Foodborne Antimicrobial Resistance in Six European Countries. *Infection and Drug Resistance* .2020;Volume 13: 10.2147/IDR.S237038957-993 10.2147/IDR.S237038 PMC714072532308439

[3] : Guidance on the Selection of Appropriate Indicators for Quantification of Antimicrobial Usage in Humans and Animals. *Zoonoses Public Health, 64(3):165–184* .2017;10.1111/zph.1229827592024

# Response

**Published:** 2026-09-13

**Authors:** Clare Chandler

**Affiliations:** London School of Hygiene & Tropical Medicine, UK

**Response to reviewer comments** Authors’ response: We thank the reviewer for the review and helpful comments. We have revised the manuscript in line with each of these suggestions in turn and believe this has strengthened the paper as a whole. **Reviewer #1. Lucie Collineau**

 Reviewer comment: This manuscript aims to provide guidance on antibiotic surveillance, specifically to support prioritization of surveillance activities within countries (or settings) with limited resources. The information provided is generally comprehensive and accurate, and the authors make an interesting point that antibiotic surveillance activities should be prioritized to feed into actions.

 Authors’ response: We thank the reviewer for their comments and summary of the article’s key points. Reviewer comment: Most of the content is not new, so I would rather classify this manuscript as a review, rather than a research article. Authors’ response: This article represents an original analysis of the guidance and tools available for the monitoring of antibiotics across systems. We recognise the limits of manuscript classification terminology, and for this journal this classification was the best fit for this article.

 Reviewer comment: The text is very long, and could be made more concise by referring to previous similar reviews, e.g. Refer to reference 1, 2 & 3 1. Babu Rajendran N, Arieti F, Mena-Benítez C, et al.: EPI-Net One Health reporting guideline for antimicrobial consumption and resistance surveillance data: a Delphi approach. *The Lancet Regional Health - Europe. *2023; **26** | Publisher Full Text 2. Mesa Varona O, Chaintarli K, Muller-Pebody B, et al.: Monitoring Antimicrobial Resistance and Drug Usage in the Human and Livestock Sector and Foodborne Antimicrobial Resistance in Six European Countries. *Infection and Drug Resistance. *2020; **Volume 13** 957-993 | Publisher Full Text 3. Collineau, L., Belloc, C., Stärk, K. D., et al.: Guidance on the Selection of Appropriate Indicators for Quantification of Antimicrobial Usage in Humans and Animals. *Zoonoses Public Health, 64(3):165–184. *2017.

 Authors’ response: We have reduced the length of the article, particularly in the Steps to Prioritisation section. We thank the reviewer for the suggested references, which we have included as they do set up the back-drop to the current paper and will be useful resources for readers.

 Reviewer comment: The authors should define the scope of “antibiotic surveillance” retained in this study; most of the content relates to surveillance of antibiotic use (and antibiotic residues, to some extent). However, surveillance of AMR in itself (and how to prioritize it) is not really covered. Maybe the title of the paper should be clarified accordingly. 

 Authors’ response: We recognise the confusion arising around the term ‘antibiotic surveillance’, raised by all reviewers, and have amended title and text to refer to antibiotic monitoring. 

 Reviewer comment: Several important initiatives are missing, such as the FAO Progressive Management Pathway for Antimicrobial Resistance (PMP-AMR), and the Quadripartite Technical Group on Integrated Surveillance on antimicrobial use and resistance (QTG-AIS).

 Authors’ response: We agree the Progressive management pathway and QJS guidance on integrated surveillance are important to include. The former is part of the inventory (see the supplementary file) and the latter is included in the text but was not yet published at the time the review for the inventory concluded.

 Reviewer comment: Description or recommendations related to environmental surveillance are rather sparse. It seems that the environmental sector was not really represented in the technical working group that worked on this study. Maybe a limitation to be mentioned in the text? Similarly, information is rather limited about AMU surveillance in plants (page 14); maybe similar tools that those used in animal farming could be used?

 Authors’ response: We note the concerns about the lack of specific guidance for plants and the environment, and suggest that this reflects the relatively lower investment in evidence and experience around the globe rather than a lack of engagement from these sectors within the working group. Given the challenges that countries face in the area of monitoring antibiotics in the environment and plants, the manuscript includes specific sections on these issues. We have clarified that at the moment there are no globally agreed standards for monitoring in these areas.

 Reviewer comment: I suggest the supplementary file that lists ongoing surveillance initiatives to focus only on global programmes. In the current version, there are a few examples from national surveillance systems as well (e.g. NARMS, German systems, etc.) , but these are nor comprehensive nor illustrative of LMIC countries, so it is a bit unclear how they were selected and why they are of interest here. They make the scope of the paper a bit fuzzy. However, examples from LMIC in the core text (as text boxes) are welcome.

 Authors’ response: We have considered the suggestion to confine the supplementary file of tools to global programmes. However, the selected inclusion of country level guidance and tools provide resources for other countries that complements and extends the case study experiences shared in the text.

 Reviewer comment: Please harmonize the terminology throughout: sector vs domain. 

 Authors’ response: We have revised the manuscript to ensure consistency in terminology and definitions throughout. We use ‘sectors’ to refer to operational spheres, such as agriculture / human health care, or the public or private care sectors. We use One Health ‘domains’ when referring to the wider spheres in which antibiotics may be distributed, used, disposed of and which may not neatly fit within an operational ‘sector.’

 Reviewer comment: Please limit the number of acronyms to the strict minimum; Page 15: what does SF means?

 Authors’ response: we have reduced acronyms as much as possible and clarified that SF means substandard or falsified medicines.

 Reviewer comment: Minor comments

 About AMR surveillance in the environment (page 11): maybe mention the new EU directive that will make AMR surveillance in wastewater mandatory in large cities (>100 000 inhab).

 Authors’ response: We have incorporated the reviewer’s suggestion to include the EU wastewater directive. 

 Reviewer comment: About engagement of local actors: not only regulation can facilitate data submission; voluntary win-win systems can be very effective and sustainable too. 

 Authors’ response: We agree with this statement and have tried to make this clearer in the text.

 Reviewer comment: Page 14: with AI and big data becoming a growing field of research, probably the data collection tools can adapt to the data sources, rather than the other way around. 

 Authors’ response: While AI and big data approaches will facilitate analysis from different data sources, we believe that standard approaches are probably even more important if data are to be analysed in a transparent way, useful and comprehensible to policy makers and comparable between sites and countries.  

 Reviewer comment: Page 15: the primary goal of AM residues monitoring is to protect the consumer from chemical risks related to residues in food (not to monitor AMU or illegal use); please edit

 Authors’ response: We have clarified the text that the goal of monitoring AM residues in food is consumer protection.

 Reviewer comment: Page 16: any other attempt than JIACRA to compare AMU between sectors?

 Authors’ response: We have provided some examples of other countries that are attempting to compare between sectors, such as Bangladesh.

 Reviewer comment: Table 1: the focus is on AMU only, please edit the title accordingly. An important gap for environmental surveillance is to select appropriate indicators; this is work in progress. Consider expanding animal health tools to plant farming? Who are the actors in charge of ‘prescribing’ AMU in plants? 

 Authors’ response: we have amended the title of Table 1 to better reflect the scope of the paper. We did not in the Table list subscribers as they can vary from country to country, both in animal and plant health systems.

 Reviewer comment: Page 18: calculate rates of usage

 Authors’ response: We think this is a suggestion to change ‘predict usage’ to ‘calculate rates of usage.’ In this case, ‘predict’ follows the term in the cited reference (Osena et al, 2026).

 Reviewer comment: Figure 3: consider adding field experts (e.g. water agencies, etc.) on environmental surveillance (not only the ministry). It seems primary care professionals are missing in this table. Not sure AMU on prescription only is feasible in certain LMIC where veterinary services are scarce.    Add NGOs and international organizations as well?

 Authors’ response: We note the considerations about which details and stakeholders should be represented in the tables and figures. While other agencies and stakeholders could be added, we are concerned not to complicate already complex figures, which are intended to be read as illustrative rather than exhaustive. 

 Reviewer comment: Page 20: add InFARM? Together with GLASS and ANIMUSE

 Authors’ response: We have added InFarm to this list.

 Reviewer comment: Table 2: consider adding pilot studies or point prevalence surveys, which are often very good starting points before building sustainable surveillance systems. They can also help to prioritize surveillance efforts by identifying high-risk populations or sectors. The way the table is presented is maybe over-ambitious (comprehensive mapping of all data sources, actors, etc.). Better start small and then expand.

 Authors’ response: We agree that point prevalence surveys are often a good place to start, but their utility is enhanced by the landscape analysis that puts them in context. The initial landscape analysis does not need to be comprehensive; as the text explains, the key thing is to estimate the relative contributions of the major productive sectors using antibiotics and the size of different components of the human health system.

 Once again, we are most grateful for the time and effort of the reviewer in assessing this manuscript and for their constructive feedback. We hope they agree the revised manuscript is much improved as a result.
